# Microbial mechanisms and therapeutic interventions in the periodontitis-inflammatory bowel disease axis: a comprehensive review

**DOI:** 10.1080/20002297.2026.2656084

**Published:** 2026-04-06

**Authors:** Wenfei Lv, Huan Hu, Yunzhuo Huang, Jieru Yang, Yanli Li, Jiyan He, Kun Wang, Yunkun Liu, Qian Wang

**Affiliations:** a Key Laboratory of Oral Disease Research of Guizhou Education Department, School of Stomatology, Zunyi Medical University, Zunyi, People's Republic of China; b Microbial Resources and Drug Development Key Laboratory of Guizhou Provincial Department of Education, School of Stomatology, Zunyi Medical University, Zunyi, People's Republic of China

**Keywords:** Periodontitis, inflammatory bowel disease, oral-gut axis, metabolism, inflammation, therapeutics

## Abstract

**Background:**

Periodontitis and inflammatory bowel disease (IBD) are chronic inflammatory conditions of the oral and gastrointestinal tracts that exhibit bidirectional microbial and immunological crosstalk.

**Objective:**

Aimed at elucidating the bidirectional crosstalk between periodontitis and IBD at both microbiological and immunological levels and evaluate related therapeutic interventions, this review comprehensively summarizes recent evidence on their interaction via the oral-gut-bone axis, focusing on microbial ecology, host responses, and innovative therapies.

**Design:**

Distinct yet overlapping dysbiotic signatures are observed in both diseases, with periodontal pathogens such as *Porphyromonas gingivalis* and *Fusobacterium nucleatum* capable of translocating to the gut and perturbing intestinal homeostasis, while gut inflammation reciprocally reshapes the oral microbiome. Mechanistic links include a spectrum of convergent pathways: (i) microbial metabolites—short-chain fatty acids, choline metabolites, indole derivatives, polyamines, and bile acids—that modulate barrier integrity and immune responses; (ii) shared immune cells and inflammatory mediators driving mucosal damage at both sites; (iii) bacterial extracellular vesicles (BEVs) and lysine lactylation (Kla)-mediated signaling; and (iv) oxidative stress, iron metabolism dysregulation, and ferroptosis contributing to tissue destruction.

**Results:**

Therapeutic strategies targeting this axis encompass bidirectional interventions: periodontal and IBD treatments that mutually influence oral and gut health, natural anti-inflammatory and antimicrobial compounds, probiotics and prebiotics, oral and fecal microbiota transplantation, and emerging bacteriophage therapy. Critically, the clinical translation of collaborative dentistry-gastroenterology management is highlighted as a promising avenue for integrated care.

**Conclusions:**

By integrating findings across microbial ecology, host response, and therapeutic innovation, this review provides a comprehensive framework for understanding and targeting the periodontitis-IBD axis.

## Introduction

**Figure 1.**
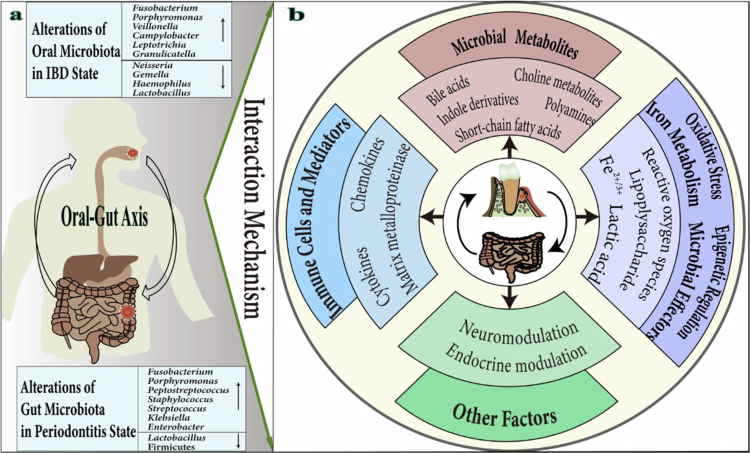
Bidirectional Microbial and Functional crosstalk in periodontitis and IBD. a. Dysbiosis along the oral-gut axis: shifts in oral microbiota observed in IBD patients (e.g. *Leptotrichia*, *Atopobium*) and gut microbiota changes in periodontitis (e.g. *Fusobacterium*, *Prevotella*). b. Key mechanisms mediating interactions between periodontitis and IBD via the oral-gut axis, including microbial metabolites (e.g. Bile acids, Choline metabolites), immune cells and mediators, oxidative stress, iron metabolism (e.g. ferroptosis), microbial effectors (e.g. BEVs) and epigenetic regulation (e.g. Kla) and other factors.

Periodontitis is a chronic inflammatory disease driven by dysbiotic dental plaque biofilms, leading to progressive destruction of tooth-supporting tissues and affecting over one billion people globally [1]. Beyond local tissue damage, periodontitis is increasingly recognised as a source of systemic inflammation, with strong epidemiological and mechanistic links to conditions such as diabetes, rheumatoid arthritis, and inflammatory bowel disease (IBD) [2,3]. Recent clinical studies further illustrate this systemic dimension: non-surgical periodontal therapy has been associated with improved arterial stiffness and endothelial function [4], while periodontal microRNAs (e.g. miR-200b-3p, miR-200b-5p) have emerged as dynamic regulators of local tissue remodelling that carry potential systemic implications [5]. Critically, periodontal pathogens can translocate to distant sites via hematogenous or alimentary routes, and growing evidence supports their role in exacerbating intestinal inflammation through immune activation along the oral-gut axis [6,7] (Table 1). IBD—encompassing Crohn’s disease and ulcerative colitis (UC)—is a chronic immune-mediated disorder of the gut, marked by recurrent intestinal inflammation and a rising global prevalence [8]. Notably, both periodontitis and IBD are characterised by microbial dysbiosis and aberrant host immune responses, suggesting potential shared pathophysiological mechanisms (Figure 1a). Accumulating clinical and experimental data now support a bidirectional relationship between these conditions, where each may influence the onset and progression of the other [3,7]. Epidemiological studies consistently report a higher prevalence and severity of periodontitis in IBD patients, supporting this bidirectional association [9,10].

**Table 1.** Major oral-derived pathobionts linking Periodontitis-IBD: mechanisms of action and supporting evidence.

Major pathogens	Mechanisms of action	Evidence source	References
*Porphyromonas* *gingivalis*	Activates FFAR2, downregulates Smad1, and inhibits osteogenic differentiation of PDLSCs	*In vitro* (human PDLSCs)	[11]
	PPAD enzyme expands Th17 cells (↑IL-17) and reduces Tregs (↓IL-10), exacerbating colitis	C57BL/6 mice, DSS-induced UC	[12]
	Secreted EVs activate FADD-RIPK1-caspase-3 signalling, impair bile acid metabolism, reduce IL-10, and disrupt gut barrier	C57BL/6 mice, DSS-induced UC	[13]
	Activates CARD3 to upregulate IL-1β, IL-6, IL-17F, and TNF-*α* via IL-17F pathway, promoting intestinal inflammation	*In vitro* (C57BL/6 mice, IEC cells)	[14]
	EVs internalised by IECs activate AIM2 inflammasome, triggering pyroptosis and barrier dysfunction	C57BL/6 mice, DSS-induced UC	[15]
	Stimulates macrophage glycolysis and H3K18 lactylation, inducing Aβ expression and activating Syk/ROS signalling	*In vitro* (Human monocytes U937)	[16]
	Promotes H4K12 lactylation and ADAM17 expression; suppresses TGF-βR1 and MerTK in macrophages, impairing osteogenesis and immune function	*In vitro* (MEPM cells)	[17]
	Induces ferroptosis via SLC7A11/GSH/GPX4 inhibition, disrupting oral epithelial barrier integrity	SD male rats, ligature/*P.gingivalis*-induced periodontitis	[18]
	FadA adhesin binds PEBP1, activating Raf1-MAPK and IKK-NF-κB pathways, increasing IL-1β, IL-6, and IL-8	*In vitro* (human PDLSCs)	[19]
	EVs induce ferroptosis in intestinal epithelium, increasing permeability	C57BL/6 mice, DSS-induced UC	[20]
*Enterococcus faecalis*	Induces macrophage apoptosis and dampens inflammation via NF-κB/MAPK modulation	*In vitro* (*E. faecalis* OG1RF ATCC 47,077)	[21]

A key mediator of this interaction is the oral-gut microbial axis. Oral microbiota, either free-living or in keratinocyte-associated complexes, typically enters the digestive tract via dietary ingestion; salivary components (water, lipids, mucins) protect these microbes and enable them to survive gastric stress and persist in the gut [7,22]. In periodontitis, this process facilitates the translocation of periodontal pathogens to the gut, where they can colonise and disrupt intestinal homoeostasis [23]. Animal models further support this link: induction of periodontitis alters gut structure and immune infiltration [24], while oral administration of saliva from periodontitis patients exacerbates colitis and enriches IBD-associated taxa such as *Blautia*, *Helicobacter*, and *Ruminococcus*[25]. Conversely, gut-derived microbial signals may influence alveolar bone metabolism via immune and metabolic pathways, suggesting a reciprocal gut-bone axis.

This review centres on the microbial and immunological mechanisms governing the crosstalk between periodontitis and IBD, encompassing pathogen translocation, dysbiosis propagation, and host responses modulated by microbial metabolites and immune mediators (Figure 1b). It further consolidates existing evidence linking the two disorders via the oral-gut-bone axis, and explores corresponding bidirectional interventions: therapeutic strategies for periodontitis and IBD that mutually shape oral and gut health, natural anti-inflammatory and antimicrobial agents, probiotics, prebiotics, oral and faecal microbiota transplantation, as well as emerging bacteriophage therapy. Ultimately, this review seeks to establish a conceptual framework for deciphering the oral-gut-bone connection and facilitating the development of coordinated management regimens for periodontitis and IBD.

## Differences and connections between periodontitis and IBD microbiota

Periodontitis and IBD share a bidirectional pathogenic relationship rooted in disrupted microbe-immune homoeostasis. Dysbiosis in one site can exacerbate inflammation at the other through microbial translocation and immune crosstalk [7]. Periodontal pathogens not only damage the intestinal epithelial barrier but also modulate host immunity, contributing to systemic inflammation [7,9]. Once translocated to the gut, these bacteria disrupt the balance between commensals and opportunistic pathogens, promoting dysbiosis [24]. Conversely, gut-derived microbes and metabolites can act as antigens, compromising oral mucosal integrity and driving autoimmune responses in the context of IBD [26]. This reciprocal inflammation-dysbiosis cycle underpins the clinical association between periodontitis and IBD (Figure 1a, b; Table 1).

### Dysbiotic microbial profiles in periodontitis and IBD

The dysbiotic microbial profiles in periodontitis and IBD share striking similarities. In periodontitis, the microbiota shifts from facultative anaerobes to Gram-negative obligate anaerobes, with enrichment of pathobionts such as *Porphyromonas gingivalis*, *Fusobacterium nucleatum*, *Tannerella forsythia*, *Treponema denticola*, *Aggregatibacter actinomycetemcomitans* and *Prevotella intermedia* [27,28]. In contrast, the gut microbiome supports barrier integrity and immune regulation under homoeostasis. In IBD, however, dysbiosis manifests as reduced diversity, increased Proteobacteria and Bacteroidetes, and decreased Firmicutes and *Lactobacillus* [29,30]. Notably, a variety of periodontal pathogens including *F. nucleatum*, *Peptostreptococcus*, *Staphylococcus* and *Streptococcus* were enriched in the colonic biopsy tissues of IBD patients, indicating niche-specific microbial alterations [31].

### Regulatory effects of periodontal pathogens on gut microecology

The regulatory effects of periodontal pathogens on gut microecology involve specific mechanisms supported by evidence from both animal models and human studies. Given the enrichment of periodontal pathogens in the saliva of periodontitis patients, these microbes can reach the gut directly or via systemic circulation following barrier disruption, thus altering gut microbial composition compared to that of healthy individuals [24,32]. In the dual mouse model of periodontitis combined with DSS treatment, oral pathobionts such as *Klebsiella* and *Enterobacter* were synchronously enriched in both the oral cavity and the intestine [33]. Gavage of salivary microbiota from periodontitis patients into mice with periodontitis-associated colitis induced marked alterations in the IBD-linked gut microbiota—specifically, reduced *Blautia* and *Helicobacter* and enrichment of *Aerococcus*—and exacerbated intestinal inflammation, whereas microbiota from healthy individuals did not elicit these effects [25]. Periodontal therapy improves gut barrier function in murine models, reducing *Alistipes*, *Barnesiella*, and *Sporobacter*, while increasing beneficial taxa such as *Turicibacter* and *Bifidobacterium* [34]. Oral pathogens including *F. nucleatum* and *Veillonella parvula* are frequently detected in the intestines of IBD patients [9,35]. Clinical studies have also demonstrated that the similarity between the gut microbiota and oral microbiota in IBD patients complicated with periodontitis is significantly higher than that in healthy controls, indicating an increased ectopic intestinal colonisation of oral bacteria in IBD patients [36]. This translocation is facilitated by gut inflammation, which creates a permissive niche for oral pathobionts, thereby establishing a feed-forward loop of dysbiosis [37]. Gastric acid suppression further promotes ectopic oral bacterial colonisation, thus accelerating IBD progression [38]. Notably, *P. gingivalis*, *F. nucleatum*, *Streptococcus* spp., and *Veillonella* spp. are enriched in the gut microbiota of IBD patients, preferentially colonise inflamed tissues, and correlate positively with disease activity, implicating them in pathogenesis [39]. For example, *F. nucleatum*, a keystone periodontal pathogen, not only promotes alveolar bone loss but also—when co-aggregated with *Enterococcus faecalis*—exhibits enhanced survival under alkaline, hyperosmotic, nutrient-deficient, and antimicrobial conditions, while significantly inducing macrophage apoptosis and attenuating pro-inflammatory responses by regulating the NF-κB/MAPK signalling pathway [21,40]. However, the role of *F. nucleatum* may be context-dependent: it exists at low levels in healthy individuals, exhibits subspecies-specific virulence, and under certain conditions may even skew immune responses toward tolerance rather than inflammation [41]. *Prevotella* spp. and *Aggregatibacter actinomycetemcomitans* have been detected in intestinal biopsies, and these pathogens show marked expansion in the guts of patients with IBD and colorectal cancer, which underscores the role of the oral-gut microbiota axis in the pathogenesis of intestinal diseases [42,43] (Figure 1a).

### Gut inflammation reshapes the oral microbiome

Conversely, IBD-associated gut inflammation reshapes the oral microbiome. Clinically, IBD patients display elevated oral-gut microbial similarity, indicating heightened susceptibility to oral dysbiosis [10,43]. In IBD patients, both salivary and subgingival microbiota show reduced levels of *Neisseria*, *Gemella*, *Haemophilus*, and *Lactobacillus*, with enrichment of *Bacteroides*, *Campylobacter*, *Fusobacterium*, *Porphyromonas*, *Veillonella*, *Leptotrichia*, *Prevotella*, *Granulicatella*, *Capnocytophaga*, and Saccharibacteria (formerly TM7); these dysbiotic changes normalise following successful IBD therapy [44–46]. This remodelling is likely mediated by systemic inflammation, altered salivary composition, and immune cell trafficking from the gut to oral mucosal sites. In murine colitis models (dextran sulphate sodium [DSS]-induced or *Citrobacter rodentium*-infected models), salivary and oral mucosal microbiota dysbiosis was consistently observed: Proteobacteria, Actinobacteria, *Klebsiella*, and *Enterobacter* were increased, whereas Firmicutes (especially *Streptococcus*) and Bacteroidetes were decreased [33,47]. These findings demonstrate bidirectional crosstalk between the oral and gut microbiomes and suggest that certain periodontal pathogens may serve as biomarkers for IBD activity (Figure 1a). This reciprocal modulation highlights the interconnectedness of oral and gut ecosystems in systemic inflammatory conditions.

## Periodontitis-IBD interactions via the gut-bone axis

**Figure 2.**
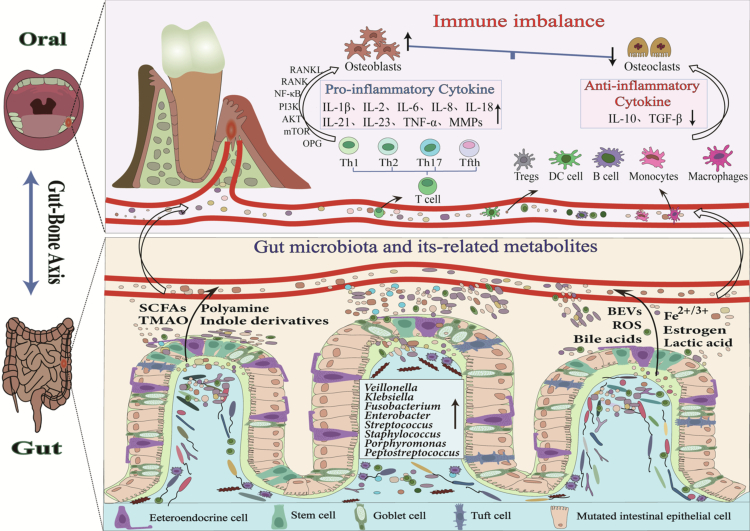
Systemic regulation of alveolar bone remodelling in periodontitis via the gut-bone axis.​​ Gut-derived and host-derived mediators—including microbial metabolites (e.g. SCFAs, bile acids), BEVs, and dysregulated host processes (e.g. iron metabolism, ferroptosis, Kla)—act systemically to disrupt osteoimmune homoeostasis. These signals converge on key signalling pathways in the periodontium, tipping the balance from osteoblast-mediated bone formation to osteoclast-driven bone resorption, ultimately leading to alveolar bone loss.

The gut microbiota plays a crucial role in regulating systemic physiology, particularly through the gut-bone axis—a signalling pathway increasingly recognised for its influence on skeletal homoeostasis, including alveolar bone integrity [9] (Figures 1b and 2). Microbial metabolites such as short-chain fatty acids (SCFAs), choline metabolites, indole derivatives, polyamines, and bile acids form a complex network that regulates host immune responses and metabolic functions. By influencing the balance between osteoclasts and osteoblasts, these gut-derived signals contribute to the regulation of alveolar bone remodelling [48]. Emerging evidence further implicates additional mediators—such as bacterial extracellular vesicles (BEVs), lysine lactylation (Kla), oxidative stress, and iron metabolism—in the modulation of this gut-bone crosstalk during periodontitis-IBD comorbidity (Table 1).

### Microbial metabolites

Microbial metabolites serve as critical mediators of the oral-gut-bone axis, linking dysbiosis in periodontitis and IBD to systemic inflammation and tissue destruction. While their molecular mechanisms and effects have been extensively characterised in vitro and in rodent models, direct clinical evidence demonstrating their causal roles in human comorbid periodontitis-IBD remains limited. The following subsections synthesise current knowledge on key metabolite classes, highlighting conserved pathways that may provide insights for future translational research.

#### SCFAs

SCFAs—primarily acetate, propionate, and butyrate—are key fermentation products of gut microbiota. They play essential roles in maintaining intestinal epithelial barrier integrity and serve as signalling molecules that modulate immune responses, metabolism, and endocrine functions [49]. SCFAs can enter the systemic circulation and exert effects on distant tissues. Butyrate enhances mineral solubility and calcium absorption by lowering luminal pH and promoting mucosal proliferation via mTOR and Wnt/β-catenin signalling, which increases villus height and epithelial surface area, thereby upregulating calcium-binding protein expression and intestinal calcium uptake [50]. In a murine model of chronic kidney disease, *Lactobacillus johnsoni*i and its metabolite cyclo (Pro-Trp) significantly reduced parathyroid hormone (PTH) levels. This suppression enhances calcium influx into intestinal epithelial cells (IECs) through calcium-sensing receptors (CaSR) and transient receptor potential vanilloid 4 (TRPV4) channels, further promoting intestinal calcium absorption [51]. Additionally, butyrate inhibits histone deacetylase (HDAC) activity, facilitating the differentiation and function of regulatory T cells (Tregs), thus contributing to immune homoeostasis [52]. In contrast, SCFAs derived from periodontal pathogens exhibit context-dependent pathogenic effects. *P. gingivalis*, *F. nucleatum*, *P. intermedia*, and *T. forsythia* produce substantial amounts of butyrate and propionate during amino acid fermentation [48]. Elevated levels of these SCFAs are detected in the gingival crevicular fluid (GCF) of periodontitis patients compared to healthy individuals, and this elevation correlates with disease severity [53]. In vitro, butyrate compromises gingival epithelial integrity, induces ROS production, and disrupts cell cycle progression in gingival fibroblasts. It also inhibits osteogenic differentiation of periodontal ligament stem cells by activating free fatty acid receptor 2 (FFAR2) and downregulating Smad1 expression [11,49]. Propionate similarly impairs adhesion, proliferation, and protein synthesis in gingival fibroblasts in vitro [48]. Collectively, these SCFAs exert cytotoxic effects on periodontal tissues, contributing to tissue destruction. Notably, non-surgical periodontal therapy significantly reduces GCF concentrations of propionate and butyrate, concurrent with clinical improvement, suggesting their potential as biomarkers of disease activity [54].

#### Choline metabolites

Choline metabolites, produced during the metabolism of choline in the body, primarily include trimethylamine (TMA), trimethylamine *N*-oxide (TMAO), and acetylcholine. TMA is produced by gut microbiota from choline and is subsequently oxidised in the liver to form TMAO, which modulates gut microbial composition, systemic immunity, and periodontal inflammation [55].

In a murine model of periodontitis, elevated hepatic interleukin-6 (IL-6) and flavin-containing monooxygenase 3 (FMO3) levels were observed, along with increased plasma lipopolysaccharide (LPS) and TMAO; notably, non-surgical periodontal treatment reduced circulating TMAO levels [56]. Experimental TMAO administration in drinking water activates the NF-κB pathway, disrupts gut microbial balance by decreasing Bacteroidetes and increasing Firmicutes, and upregulates osteoclast-related genes via the NF-κB/NFATc1 signalling axis, ultimately promoting alveolar bone loss [57]. This dysbiosis further enhances TMAO production, creating a feed-forward loop that exacerbates inflammation and bone resorption. Gold nanospheres can significantly reduce the relative abundance of Firmicutes and increase the relative abundance of Proteobacteria, thereby lowering TMAO levels and reducing the production of pro-inflammatory cytokines (e.g. IL-6 and TNF-*α*), thus regulating bone metabolism [58].

#### Indole derivatives

Indole derivatives, microbial metabolites of tryptophan, enhance antimicrobial peptide and mucin production, stimulate intestinal epithelial proliferation, and suppress pathogen expansion, thereby preserving gut barrier integrity. Dietary indole-3-propionic acid (IPA) in rat pups activates the PXR/ACBP pathway, modulates the gut microbiota, and upregulates tight junction proteins, mucins, and interleukin-10 (IL-10) in IECs, while suppressing IL-8 expression [59]. These changes strengthen the intestinal barrier and may indirectly influence bone homoeostasis.

5-Hydroxytryptamine (5-HT), a key indole derivative, exerts opposing effects on alveolar bone remodelling based on its site of action. Central 5-HT activates calmodulin-dependent kinases (CaMKKβ/CaMKIV), leading to CREB phosphorylation, reduced sympathetic nervous system activity, and enhanced osteogenesis [60]. In contrast, peripheral 5-HT stimulates IL-6 secretion in bone cells via 5-HT_2_B and ERK1/2 signalling, which in turn promotes osteoclastogenesis and bone resorption [61]. Thus, gut-derived indole derivatives may impact periodontal health through the gut-bone axis and direct regulation of alveolar bone turnover.

#### Polyamines

Polyamines are essential molecules involved in cell growth, proliferation, differentiation, and survival and are synthesised and released by the gut microbiota [62]. For example, putrescine produced by *Escherichia coli* in the mouse colon is absorbed by IECs and subsequently converted to spermidine, which accelerates epithelial renewal and increases the population of anti-inflammatory macrophages in the colon, thereby contributing to gut homoeostasis [63]. In obese or type 2 diabetic mice, dietary supplementation with polyamines promotes the polarisation of macrophages toward an anti-inflammatory phenotype, reduces the levels of pro-inflammatory cytokines (e.g. TNF-*α* and IL-6), and upregulates the expression of IL-10—effects that help restore systemic immune homoeostasis [64]. Furthermore, polyamines can regulate intestinal flora, repair the intestinal barrier, inhibit the differentiation and maturation of osteoclasts, and reduce bone loss in the weightlessness-induced bone loss (WIBL) model, suggesting that they exert a protective effect against pathological bone resorption [65]. Given that periodontitis-associated alveolar bone loss is also driven by chronic inflammation and excessive osteoclast activity, polyamines may similarly protect against periodontal tissue destruction through systemic immunomodulation.

#### Bile acids

Bile acids are signalling molecules that regulate lipid metabolism, bone homoeostasis, and energy balance; they can also inhibit the overgrowth of the gut microbiota [66]. They exist in two forms: 1) Primary bile acids: Synthesised in the liver as bile salts and subsequently secreted into the small intestine; 2) Secondary bile acids: Generated via gut microbiota metabolism of primary bile acids, such as 3-oxolithocholic acid (3-oxoLCA), they can directly bind to the RORγt receptor, inhibiting Th17 cell differentiation. Isolithocholic acid (isoLCA) can promote Treg differentiation by inducing mitochondrial ROS levels, thereby participating in the regulation of bone metabolism and affecting alveolar bone health [67].

### Immune cells and mediators in oral-gut inflammatory crosstalk

#### Role of immune cells in periodontal and IBD-associated intestinal inflammation

In the context of periodontitis and IBD, immune cell trafficking from oral-draining lymph nodes to the intestine occurs under physiological conditions, establishing a cellular basis for oral-gut immune crosstalk [9]. Shared immunoinflammatory mechanisms between these two chronic inflammatory diseases enable their bidirectional interaction. In experimental periodontitis-colitis models, periodontal pathogens activate oral-derived CD4⁺ T cells expressing the gut-homing integrin α_4_β_7_, which migrate to and accumulate in inflamed intestinal tissues; in contrast, homoeostatic gut microbiota do not elicit this response [33]. The ectopic colonisation of periodontal pathogens (e.g. *P. gingivalis*) in the gut enables their secreted virulence factor—peptidylarginine deiminase (PPAD)—to exacerbate intestinal inflammation by increasing the number of Th17 cells and the production of IL-17, while reducing the count of Tregs and the secretion of IL-10 in the spleen of UC mice [12].

Strains isolated from CD patients exhibit higher invasiveness and more potently upregulate MUC2 and TNF expression compared to those from healthy individuals [68]. Atarashi et al. [69] demonstrated that *Klebsiella pneumoniae* isolated from the salivary microbiota of CD patients activates dendritic cells (DCs) and epithelial cells through the Toll-like receptor 4 (TLR4) signalling pathway, inducing IL-18 secretion, Th1 cell recruitment, and subsequent intestinal inflammation. *F. nucleatum* binds inhibitory receptors to suppress natural killer cell cytotoxicity and survives within macrophages to evade lymphocyte-mediated clearance, while its virulence factors—including outer membrane proteins Fap2 and RadD—inhibit T cell function and induce lymphocyte apoptosis [70]. Paradoxically, it also promotes CD4⁺ T cell differentiation into pro-inflammatory Th1 and Th17 subsets, thereby exacerbating experimental colitis in mice [71].

Gut dysbiosis further disrupts immune homoeostasis by suppressing Treg differentiation, promoting Th17 polarisation, expanding the pool of osteoclast precursors, and enhancing RANKL expression in stromal cells, collectively facilitating osteoclastogenesis and bone resorption [72,73]. Neutrophils are central effector cells in periodontal tissue destruction. Peripheral neutrophils from patients with active IBD exhibit significantly elevated metabolic activity compared to those from patients with inactive IBD, who in turn display higher activity than neutrophils from healthy controls [74]. Notably, *Wolinella* species isolated from the periodontal microbiota of IBD patients can potently modulate neutrophil chemotaxis in vitro, suggesting a functional link between periodontal dysbiosis and systemic innate immune dysregulation [75,76].

#### Role of immune mediators in periodontal and IBD-associated intestinal inflammation

Periodontitis and IBD, two chronic inflammatory disorders, share common immune mediators, which lay the foundation for their bidirectional crosstalk. Periodontal pathogen virulence factors trigger adaptive immune responses in the oral cavity, activating periodontal ligament fibroblasts, gingival fibroblasts, and infiltrating inflammatory cells to sustain inflammation; these cells secrete pro-inflammatory mediators, including cytokines, chemokines, and matrix metalloproteinases (MMPs), and upregulate osteoclastogenic genes, driving tissue destruction (Figure 2). Key cytokines such as TNF-*α*, IL-1β, and IL-6 directly promote periodontal tissue degradation and bone resorption and serve as biomarkers of disease severity [7,9,76]. Furthermore, *F. nucleatum* can modulate the activation of caspase recruitment domain 3 (CARD3) to upregulate the expression of pro-inflammatory cytokines (including IL-1β, IL-6, IL-17F, and TNF-*α*) and induce intestinal inflammation via the IL-17F signalling pathway [13,14]. Notably, elevated levels of IL-1β, IL-6, IL-21, sCD40L, IL-23, and IFN-*γ* in the periodontium correlate with the severity of IBD-associated intestinal inflammation, supporting the existence of an oral-gut inflammatory axis [37].

In DSS-induced colitis models, TNF-*α*, IL-6, and IL-1β are upregulated—findings that underscore the central role of TNF-*α* in IBD pathogenesis [72,73,77]. Clinically, anti-TNF-*α* monoclonal antibodies reduce inflammation and promote mucosal healing [78]. IL-6 signalling via its receptor drives inflammation, and IL-6R blockade inhibits leucocyte recruitment and ameliorates colitis in preclinical models [79]. TNF-*α* also stimulates MMP secretion, and these proteases contribute to the pathogenesis of both periodontitis and IBD [37]. Elevated levels of TNF-*α*, IL-2, IL-6, and IL-8 in IBD patients correlate with disease activity, and the combined detection of these cytokines may facilitate the early diagnosis and assessment of disease progression [9,79].

### BEVs

BEVs are 50-250 nm spherical nanostructures secreted by bacteria, encapsulating LPS, phospholipids, peptidoglycan, outer membrane proteins, enzymes, and signalling molecules [80]. Their nanoscale size and bilayer membrane confer resistance to degradation and enable systemic dissemination. BEVs often exhibit greater pathogenicity than their parent bacteria, penetrating deep tissues and potently activating host inflammatory pathways [81]. These distinct features enable BEVs to act as potent vectors mediating the crosstalk between periodontitis and IBD. Periodontal pathogen-derived BEVs (e.g. *Filifactor alocis* BEVs) contribute to biofilm formation and, upon binding to immune cell receptors, activate inflammatory signalling, induce osteoclast differentiation, and drive alveolar bone loss, thereby accelerating periodontitis progression [82]. Critically, BEVs deliver virulence factors to distant sites; they can be internalised by intestinal epithelial cells and initiate gut inflammation via immune activation [13,15,83].

In IBD patients, translocation of periodontal pathogens alters gut microbiota composition and shifts the gut-derived BEV profile—increasing BEVs derived from Proteobacteria and decreasing those from Firmicutes [84]. Pathogenic enteric bacteria (e.g. *E. coli*, *Pseudomonas aeruginosa*) produce BEVs containing cytotoxins that disrupt protein synthesis, trigger macrophage mitochondrial apoptosis, and stimulate pro-inflammatory cytokine production [85]. Conversely, *Bifidobacterium fragilis* and *Bacteroides thetaiotaomicron* BEVs modulate bone metabolism indirectly by influencing SCFA and enzyme metabolism in intestinal cells [86], while *Lactobacillus animalis* BEVs promote bone formation by enhancing endothelial cell activity, stimulating mesenchymal stem cell osteogenic differentiation, and facilitating angiogenic-osteogenic paracrine signalling [87]. Notably, *Akkermansia muciniphila* BEVs exert systemic osteoprotective effects, accumulating in bone tissue and rebalancing bone remodelling through enhanced osteoblast activity and suppressed osteoclastogenesis in osteoporotic models [88]. While these findings highlight the potential of BEVs as therapeutic targets, direct clinical evidence in human patients with periodontitis or IBD remains limited.

### Kla

Lactic acid, a central glycolytic metabolite, functions as both an energy substrate and a signalling molecule [89]. Its signalling role is largely mediated through Kla, a novel post-translational modification where lactyl groups are covalently attached to lysine residues, thereby modulating gene expression and cellular function [90]. Kla serves as a key link between microbial activity and host responses, offering novel therapeutic directions for periodontitis and IBD.

Periodontitis patients exhibit elevated lactate levels in GCF, which normalise after treatment, paralleled by increased Kla levels in periodontal epithelial and inflammatory cells [91]. High lactate concentrations (e.g. 10 mmol/L) can upregulate the expression of integrin α5, IL-6, IL-8, adhesion molecules, and RANKL in periodontal tissues, damaging junctional epithelial cells [92]. Furthermore, *P. gingivalis* infection stimulates macrophage glycolysis and H3K18la modification, induces the expression of amyloid-*β* (Aβ), which in turn triggers the Syk/ROS signalling pathway, thereby accelerating osteoclastogenesis and bone loss [16,93]. Transgenerationally, maternal *P. gingivalis* can promote glycolysis and the expression of H4K12la and ADAM17 in offspring. This reduces TGF-*β* receptor 1 in mouse embryonic palatal mesenchymal cells (MEPM) and Mer tyrosine kinase in macrophages, consequently inhibiting osteogenic differentiation and macrophage function, and leading to aberrant bone formation [17].

In colitis, B-cell adaptor for phosphoinositide 3-kinase (BCAP)-deficient macrophages display impaired glycolysis, resulting in reduced lactate production, diminished histone Kla levels, and suppressed tissue-repair gene expression [94]. Exogenous lactate supplementation restores Kla levels and promotes the transition of macrophages from a pro-inflammatory to a reparative phenotype. At inflammatory sites, lactate also reprograms Th17 cells via a ROS-dependent mechanism that suppresses IL-17A and enhances Foxp3 expression, thereby driving Th17-to-Treg conversion and ameliorating colitis [95]. Gut-derived lactate from *Saccharomyces cerevisiae* further attenuates inflammation by reducing IL-6/IL-1β production, inhibiting M1 macrophage polarisation, and elevating H3K18la levels [96]. Collectively, these findings establish Kla as a critical metabolic-immune interface linking microbial activity to host responses, revealing novel therapeutic targets for periodontitis and IBD.

### Oxidative stress

Oxidative stress, characterised by an imbalance between ROS production and antioxidant defenses, leads to excessive ROS accumulation and can exacerbate both periodontitis and IBD. In periodontitis, neutrophils recruited to periodontal tissues release ROS to clear pathogens but also induce oxidative stress, promoting inflammasome formation, gingival fibroblast apoptosis, and inhibition of periodontal ligament cell activity [97]. Clinically, periodontal disease patients exhibit elevated salivary biomarkers such as malondialdehyde (MDA) and 8-hydroxy-2'-deoxyguanosine (8-OHdG), which correlate with disease severity and bleeding on probing [98]. Excessive ROS not only stimulate matrix metalloproteinases (MMPs) release at infection sites but also upregulate the RANKL/OPG ratio, thereby promoting osteoclast differentiation and alveolar bone resorption. Antioxidants significantly alleviate alveolar bone resorption and attachment loss in rat models of periodontal disease [99].

Similarly, gut microbiota dysbiosis drives persistent ROS generation, disrupting systemic redox balance and causing oxidative damage that compromises intestinal cell homoeostasis and microbial diversity [100]. Colonic enterococci can rapidly generate substantial amounts of substantial ROS, inducing localised oxidative stress and illustrating shared oxidative mechanisms linking oral and gut pathologies. Under the dual effects of gut microbiota disruption and oxidative stress, intestinal mucosal damage and inflammation propagate through a self-perpetuating cycle involving goblet cell depletion, crypt hyperplasia, reduced mucus production, and ulceration. This compromises epithelial barrier function and facilitates pathogen penetration, thereby exacerbating IBD [100]. Antioxidants have shown promise in alleviating intestinal mucosal damage and improving outcomes in IBD patients.

Ye et al. [101] demonstrated that nano-selenium particles combined with Eucommia ulmoides (EUP-SeNP) inhibit the activation of the TLR4/NF-κB signalling pathway, enhancing colon antioxidant capacity and regulating gut microbiota composition, thus alleviating DSS-induced colitis in mice. Probiotics like engineered *Lactobacillus lactis* directly target inflammatory sites by releasing superoxide dismutase (SOD) to scavenge ROS and reduce oxidative damage [102]. These findings support the therapeutic potential of combined antioxidant-probiotic formulations for managing both oxidative stress and microbial dysbiosis in IBD and periodontitis.

### Iron metabolism and ferroptosis

Iron is essential for oxygen transport, storage, and cellular energy metabolism, and its metabolic dysregulation can exacerbate both periodontitis and IBD. Physiologically, Fe³⁺ is internalised via transferrin receptor (TFRC)-mediated endocytosis, reduced to Fe²⁺ by STEAP3, and transported into the cytosol by DMT1 for use in enzymes or storage in ferritin, maintaining intracellular iron homoeostasis [103]. Dysregulation of iron metabolism leads to Fe²⁺ accumulation, driving Fenton reactions that generate ROS and cause oxidative damage to lipids, proteins, and DNA, ultimately inducing ferroptosis [104].

Patients with gingivitis or periodontitis exhibit elevated iron levels in gingival crevicular fluid and serum [105]. Periodontal pathogens disrupt iron homoeostasis by: (i) cleaving iron-binding proteins to release free iron; (ii) inducing erythrocyte lysis; and (iii) inhibiting osteoblast function via iron-mediated pathways [106]. *P. gingivalis* induces ferroptosis by inhibiting the SLC7A11/GSH/GPX4 axis, thereby disrupting the oral mucosal barrier integrity [18]. Concurrently, periodontitis impairs antioxidant defenses, depleting glutathione (GSH) and increasing susceptibility to lipid peroxidation (LPO) and ferroptosis [107]. Glutathione peroxidase 4 (GPX4), a key ferroptosis inhibitor, shows reduced activity in periodontitis, which in turn exacerbates the release of inflammatory cytokines (TNF-*α*, IL-6, IL-1β, IL-18) [108]. *F. nucleatum* infection in human periodontal ligament stem cells (PDLSCs) suppresses proliferation, promotes apoptosis and ferroptosis, and upregulates iron metabolism genes (e.g. *Ferritin*) while downregulating antioxidant genes (e.g. *GPX4*), thus linking microbial challenge to ferroptotic damage [19]. In colitis models, *F. nucleatum* activates the ferroptosis pathway to disrupt the intestinal barrier—consistent with its pathogenic role in periodontal tissues—characterised by elevated Fe²⁺ and MDA levels, glutathione depletion, dysregulated *GPX4*/*FTH1*/*ACSL4* expression, reduced mitochondrial membrane potential, and ROS accumulation in mouse colonic tissues [20].

In both colitis models and IBD patients, ferroptosis markers are elevated, including *PTGS2* and LPO indicators (ROS, COX2, *ACSL4*), alongside reduced GPX4 expression and impaired NF-κB p65 phosphorylation—normally protective against endoplasmic reticulum (ER) stress-induced ferroptosis [109,110]. Iron chelators reduce intestinal ROS and disease severity, highlighting their therapeutic potential [111]. High dietary iron intake increases IBD risk by reducing SCFA production, impairing probiotic growth, and promoting macrophage iron accumulation during infections (e.g. with *B. fragilis*) [112,113]. Thus, iron dysregulation represents a mechanistic link between periodontitis and IBD, with ferroptosis regulators emerging as promising therapeutic targets.

### Other factors

Emerging evidence implicates endocrine dysregulation, neuro-immune crosstalk, and genetic susceptibility in the shared pathogenesis of periodontitis and IBD, offering new insights into their bidirectional relationship. Collectively, these factors contribute to the complex interplay between periodontitis and IBD (Figure 2).

Estrogen deficiency promotes alveolar bone resorption by activating the RANKL/RANK/OPG pathway and impairing periodontal tissue homoeostasis [114]. It also disrupts gut microbiota composition, reducing beneficial *Bifidobacterium* and *Lactobacillus* while enriching pro-inflammatory Enterobacteriaceae, thereby amplifying intestinal inflammation via TNF-*α* and IL-1β [115]. Similarly, parathyroid hormone (PTH)-driven bone loss involves gut microbiota-dependent Th17 activation, with Segmented Filamentous Bacteria enhancing TNF production and osteoclastogenesis [116].

Neuroendocrine pathways, including the brain-gut-bone axis, regulate skeletal integrity. Dopamine promotes osteoblast activity via the Wnt/β-catenin signalling pathway; leptin modulates bone metabolism through hormonal cross-talk; and serotonin influences bone homoeostasis via corticotropin-releasing hormone (CRH) regulation [117]. In Parkinson’s disease models, systemic LPS elevation activates the TLR4/MyD88/NF-κB signalling pathway in the gut and brain, triggering inflammation that secondarily impairs bone homoeostasis [118].

Genetic polymorphisms in immune sensors like TLRs and nucleotide-binding oligomerization domain 2 (NOD2) increase susceptibility to both diseases. NOD2 variants impair recognition of microbial components (e.g. muramyl dipeptide [MDP] from *P. gingivalis*), leading to defective host responses in periodontitis and disrupted gut immune homoeostasis in IBD—including reduced antimicrobial peptide production and compromised pathogen containment [74,118,119]. This shared genetic vulnerability positions NOD2 as a molecular link between oral and intestinal inflammation, suggesting its pathway as a potential dual-target therapeutic strategy.

## Therapeutic modulation of the microbiome in periodontitis and IBD

**Figure 3.**
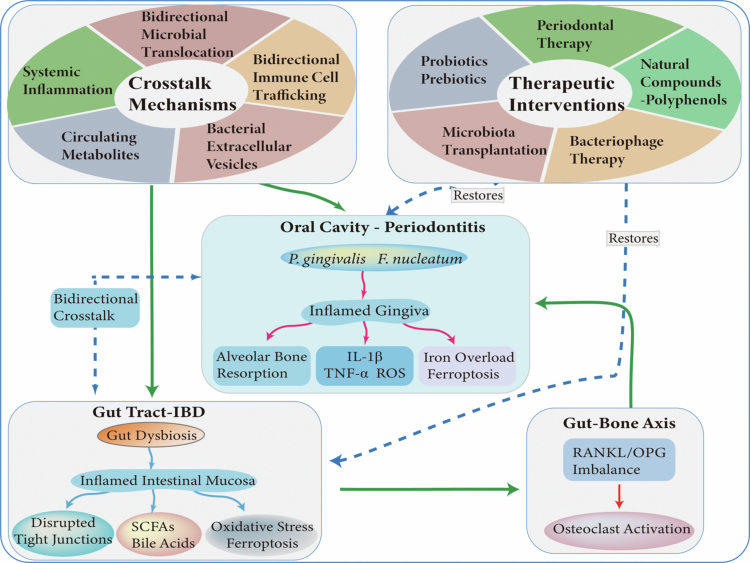
Bidirectional Crosstalk Between Periodontitis and IBD via the Oral-Gut Axis: Therapeutic Strategies and Implications for Bone Metabolism. The core crosstalk mechanisms illustrate key pathological signalling pathways linking the oral cavity and gut. Therapeutic strategies aim to restore oral-gut microbial and immune homoeostasis, interrupt inflammatory cascades, and thereby ameliorate comorbid outcomes in periodontitis and IBD, including alveolar and systemic bone loss.

Emerging microbiome-targeted strategies—including probiotic and prebiotic supplementation, faecal microbiota transplantation (FMT), and dietary interventions—hold therapeutic promise for both periodontitis and IBD. Clinical evidence indicates that specific probiotic strains (e.g. *Bifidobacterium* and *Lactobacillus* spp.) modulate oral-gut microbial crosstalk and fine-tune local immune responses [120]. These approaches have been translated into diverse formulations, including oral tablets, functional beverages, and topical agents (e.g. mouthwashes and toothpastes), laying the groundwork for integrated, system-wide therapies focused on microbiome regulation (Figure 3; Table 2).

**Table 2.** Therapeutic Interventions Targeting the Periodontitis-IBD Axis: Modalities, Mechanisms, and Evidence Levels.

Modalities	Target site(s)	Mechanism of action	Model system	References
Quercetin	Oral	Activates NRF2 in PDLSCs, reduces oxidative stress, and alleviates alveolar bone loss	C57BL/6 mice, Ligature-induced periodontitis	[121]
	Oral	Inhibits *P. gingivalis* virulence (gingipains, hemagglutinins) and biofilm formation	*In vitro* (*P. gingivalis* ATCC 33277)	[122]
	Oral/Gut	Downregulates miR-147-5p to promote Clip3 activation, suppressing Th17 differentiation and alveolar bone repair	SD rats, Ligature-induced periodontitis	[123]
Baicalin	Oral/Systemic	Restores Firmicutes/Bacteroidetes ratio, enriches SCFA-producing taxa, and ameliorates alveolar bone loss and gut barrier dysfunction	C57BL/6 mice, Ligature-induced periodontitis	[124]
	Gut	Modulates bile acid metabolism and suppresses NF-κB/PPARα signalling to reduce mucosal inflammation	C57BL/6 mice, DSS-induced UC	[125]
*Lacticaseibacillus rhamnosus* GG	Oral/Gut/Systemic	Improves alveolar bone parameters, modulates gut microbiota, and elevates selenomethionine to promote osteogenesis	SD rats, Periodontitis with hyperlipidemia	[126]
*Clostridium butyricum*	Gut	Enhances tight junctions, increases goblet cells and MUC2, and reduces pro-inflammatory cytokines	C57BL/6 mice, DSS-induced UC	[127]
*Escherichia coli* NISSLE 1917	Gut	Scavenges ROS, down-regulate pro-inflammatory cytokine levels, regulates gut microbiota, and repairs epithelial barrier	C57BL/6 mice, DSS-induced UC	[128]
Inulin	Gut	Scavenges ROS, down-regulate pro-inflammatory cytokine levels, and promote epithelial barrier repair	SD rats or C57BL/6 mice, DSS-induced UC or TNBS-induced CD	[129]
Faecal Microbiota Transplantation (FMT)	Gut	Reduces *Eubacterium hallii* and *F. nucleatum* enrichment, enhances SCFAs synthesis and secondary bile acid levels	Human, double-blind RCT in UC patients	[130]
	Gut	Downregulates the peroxidase family, increases IL-10 secretion, and elevates the abundance of SCFA-producing and secondary bile acid-producing bacteria	C57BL/6 mice, DSS-induced UC	[131]
	Gut	Promotes Foxp3⁺/RORγt⁺ Tregs expansion, induces IL-10 and SCFAs production, suppresses mucosal inflammation	C57BL/6 mice, DSS-induced UC	[132]
	Gut	Upregulates occludin and claudin-1, reduces the proportion of CD4⁺RORγt⁺ Th17 cells, and increases the proportion of CD4⁺Foxp3⁺ Treg cells	Human, clinical trial	[133]

### Bidirectional effects of periodontal and IBD treatments on intestinal and oral health

Periodontal therapy not only targets local oral biofilms but also exerts systemic effects on gut homoeostasis. The expression of periodontitis-related biomarkers exhibits stage-specific dynamic evolutionary characteristics, which are closely correlated with disease progression, treatment response and prognostic outcome [28]. The active phase of the disease is characterised by abnormally elevated levels of pro-inflammatory factors and tissue degradation markers (e.g. TNF-*α*, IL-6, IL-1, ROS, and MMPs), reflecting active periodontal tissue destruction and intense inflammatory responses [7,9]. Following standardised periodontal supportive therapy, levels of pro-inflammatory markers decrease significantly, while anti-inflammatory and repair-regulating factors (e.g. IL-10 and Tregs) tend to increase or restore balance, leading to a restoration of immune homoeostasis. Concurrently, clinical parameters including bleeding on probing, probing depth, clinical attachment level, and full-mouth plaque index shift toward a healthy status [34,45]. Notably, recent evidence highlights that the efficacy of such care is protocol-dependent. Supportive Periodontal Care (SPC) incorporating subgingival instrumentation yields superior outcomes in reducing bleeding on probing compared to supragingival cleaning alone, although deep pockets and smoking habits remain negative predictors of treatment response [134]. Furthermore, adjunctive chemical therapies are being optimised; for instance, chlorhexidine formulations supplemented with sodium DNA maintain antimicrobial efficacy against oral bacteria while significantly attenuating host cytotoxicity and metabolic stress, offering a refined strategy to control dysbiosis without compromising tissue compatibility [135]. Scaling and root planing (SRP), the gold-standard treatment for periodontitis, mechanically removes subgingival biofilm, calculus, and bacterial toxins, and is often combined with local antimicrobials (e.g. metronidazole, chlorhexidine, minocycline) to enhance efficacy. In preclinical models, hyperlipidemic apolipoprotein E-deficient (apoE⁻/⁻) mice with periodontitis treated with SRP showed restored intestinal barrier function and enrichment of butyrate-producing taxa, accompanied by reduced abundances of *Alistipes*, *Barnesiella*, and *Sporobacter* and increased abundances of *Turicibacter* and *Bifidobacterium*[34]. Consistent with these findings, clinical studies demonstrate that periodontal therapy induces significant remodelling of the gut microbiota in patients, including increased Actinobacteria and reduced Bacteroidetes and Verrucomicrobia; in advanced cases, decreases in *Bacteroides*, *Faecalibacterium*, and *Lachnospiraceae* are observed [136].

Conversely, systemic IBD therapies can modulate oral inflammation and immunity. Standard pharmacotherapy for IBD includes aminosalicylates, corticosteroids, immunosuppressants, and biologics such as anti-TNF antibodies, anti-IL-12/23 agents, and leucocyte trafficking inhibitors [137]. Although direct mechanistic studies in animal models of periodontitis are limited, human evidence indicates that anti-TNF-*α* agents confer ancillary benefits on oral health: IBD patients with periapical periodontitis treated with these biologics exhibit accelerated healing compared to those receiving dental treatment alone, along with elevated salivary IgA and myeloperoxidase (MPO) levels [98].

Together, these findings indicate that interventions targeting either condition exert reciprocal regulatory effects across the oral-gut axis: periodontal therapy improves gut microbial composition and mucosal integrity, while IBD treatment ameliorates oral inflammation. This bidirectional benefit underscores the potential for integrated clinical management. Nevertheless, robust interventional trials are still lacking—particularly those assessing whether periodontal treatment improves IBD activity or vice versa—warranting further investigation. In the meantime, routine oral health monitoring remains essential for IBD patients, as oral inflammation may serve as an indicator of systemic disease activity.

### Natural compounds with anti-inflammatory and antimicrobial activities in periodontitis and IBD

Natural bioactive compounds show promise in modulating inflammation and microbial dysbiosis in both periodontitis and IBD [138]. Quercetin inhibits *P. gingivalis* virulence factors, including gingipains and hemagglutinins, and activates the Nrf2 pathway in oxidatively stressed PDLSCs, enhancing antioxidant defenses and osteogenic differentiation [121,122]. In murine periodontitis models, quercetin suppresses pro-inflammatory mediators (IL-1β, TNF-*α*, IL-17), miR-147-5p/Clip3 axis, and osteoclastogenic signalling (RANKL), significantly attenuating alveolar bone resorption [123].

Baicalin exhibits broad antimicrobial activity against periodontal pathogens such as *S. salivarius*, *P. gingivalis*, *A. actinomycetemcomitans*, and *F. nucleatum*, and reduces IL-1β-induced PGE₂ production, thereby mitigating periodontal inflammation and bone loss [124]. In IBD models, baicalin normalises the Firmicutes/Bacteroidetes ratio, enhances SCFA-producing taxa, and restores intestinal barrier function. When combined with magnesium, it improves solubility, modulates bile acid metabolism, and suppresses NF-κB and PPARα signalling, reducing oxidative stress and mucosal damage [125]. In addition, a nanocomposite of carboxymethyl chitosan, infliximab (IFX), and chondroitin sulphate (CS) resists gastric enzymatic degradation and pH variability, enhancing intestinal drug availability [139]. Notably, this nanocomposite can be co-delivered with natural compounds to synergistically improve therapeutic efficacy.

Conventional drugs face limitations including poor bioavailability, drug resistance, and systemic toxicity, prompting the development of advanced delivery systems to improve solubility, stability, and targeted tissue-specific delivery. Given that blind use of antibiotics may disrupt the balance of intestinal symbiotic flora, exacerbate dysbiosis, and conversely promote the growth and colonisation of pathogenic bacteria such as *P. gingivalis* and *F. nucleatum*, future therapeutic strategies are likely to combine natural compounds with both anti-inflammatory and antibacterial activities with novel drug delivery systems to achieve targeted therapy; nevertheless, this strategy remains in the experimental stage. It is worth noting that further studies are needed to clarify the optimal combination ratios and safety profiles of these natural compounds—as well as their synergistic mechanisms with drug delivery systems.

### Probiotics and prebiotics in periodontitis and IBD

Probiotics and prebiotics can ameliorate IBD and exert osteoprotective effects via the gut-bone axis. Probiotic supplementation is an emerging adjunct to standard periodontal therapy. In hyperlipidemic rats with periodontitis, administration of *Lacticaseibacillus rhamnosus* (*LGG*) improved alveolar bone parameters—including bone mineral density (BMD), bone volume (BV), and trabecular thickness (Tb.Th)—and increased gut abundance of *Staphylococcus*, *Corynebacterium*, and *Collinsella*. FMT from *LGG*-treated donors to diseased recipients elevated levels of osteogenic metabolites such as selenomethionine (SeMet), promoting osteogenic differentiation of bone marrow mesenchymal stem cells [126]. Clinical studies demonstrate that probiotics significantly improve outcomes of SRP, enhancing clinical attachment level (CAL), reducing bleeding on probing (BOP), and improving gingival and plaque indices [140].

Probiotic, prebiotic, and synbiotic interventions modulate gut microbiota and inflammation in IBD, offering safe adjunctive therapy. *Clostridium butyricum* enhances intestinal barrier function in murine colitis by strengthening tight junctions, increasing goblet cells and MUC2 production, and shifting the Firmicutes/Bacteroidetes ratio—collectively attenuating inflammation [127,141]. Engineered probiotics, such as CS-sodium alginate-coated ECN-pE(C/A)₂, improve gastrointestinal survival, colonise efficiently, scavenge ROS via catalase and SOD expression, suppress pro-inflammatory cytokines (IL-1β, TNF-*α*), enhance anti-inflammatory mediators (IL-10, TGF-*β*), and upregulate ZO-1 and occludin to restore barrier integrity, while enriching SCFA-producing taxa like *Lachnospiraceae* NK4A136 [128]. Prebiotic composites, including olsalazine-inulin gels, modulate microbial ecology, reduce Proteobacteria expansion, and promote epithelial repair [129]. Emerging evidence indicates that these microbiome-targeted interventions exert systemic effects beyond the gut. Certain probiotics (e.g. *L. reuteri* ATCC PTA 6475) improve bone homoeostasis by correcting dysbiosis and reducing systemic inflammation [142], while others may inhibit bone resorption through metabolic modulation [143,144]. Prebiotics like inulin and fructooligosaccharides (FOS) enhance microbial diversity and SCFA production, contributing to reduced inflammation and bone loss [145].

These findings indicate that probiotics exert systemic benefits beyond the oral cavity, potentially mediating periodontal regeneration through gut microbiota-dependent metabolic signalling. Specifically, probiotics and prebiotics exhibit orally relevant systemic anti-inflammatory and osteoprotective potential by modulating the gut-bone axis. Although direct effects on alveolar bone resorption require further validation, the established role of gut microbiota in regulating systemic bone metabolism suggests potential benefits for periodontal bone preservation in IBD patients. Notably, probiotics and prebiotics have already been clinically applied in the treatment of both diseases, and their continued development is expected to become an important research direction for the future therapy of periodontitis and IBD.

### Oral and faecal microbiota transplantation

Microbiota transplantation can regulate microbial homoeostasis at both the oral and gut sites, demonstrating promising translationalapplication prospects. Oral microbiota transplantation (OMT) involves transferring health-associated oral microbial communities to restore microbial balance and suppress periodontal pathogens [146]. Preclinical studies in beagle dogs show that OMT significantly increases oral alpha diversity, reduces periodontal inflammation, and improves clinical outcomes compared to mechanical plaque removal alone [147]. While human trials are currently lacking, these findings support OMT as a promising strategy for patients unresponsive to conventional therapies, offering targeted microbiome restoration as a future direction in periodontitis management [148].

FMT has emerged as a clinically validated intervention for modulating gut microbiota composition and function, demonstrating significant efficacy in improving both clinical symptoms and endoscopic remission rates in IBD patients [149]. FMT promotes engraftment of beneficial taxa such as *Faecalibacterium prausnitzii* and *Roseburia inulinivorans*, enhancing SCFA and secondary bile acid production, while improving mucus metabolism and butyrate synthesis [130,131,150]. Its immunomodulatory effects include rebalancing intestinal T cell subsets—reducing CD4⁺ T cell abundance and promoting Tregs differentiation via strains like *Bacteroides visceratus*, which induce IL-10 secretion and suppress mucosal inflammation [132,133]. These systemic changes in microbial and immune homoeostasis suggest potential indirect benefits for extra-intestinal inflammatory conditions, including periodontitis.

Future therapeutic regimens may be designed to integrate natural compounds with both anti-inflammatory and antibacterial activities, along with probiotics or microbiota transplantation via novel targeted drug delivery systems. By modulating the microbial community balance at both ends of the oral-gut axis, such strategies aim to break the vicious cycle between chronic inflammation and dysbiosis; however, this requires further clinical validation.

### Bacteriophage therapy

Bacteriophage therapy has emerged as a promising experimental strategy for IBD due to its high specificity for pathobionts such as adherent-invasive *Escherichia coli*, enabling their selective elimination while preserving commensal microbiota [151]. Advanced delivery systems, including dextran-based nanoparticles, enhance phage stability and targeting efficiency, potentially extending activity to other pro-inflammatory species like *F. nucleatum*—a bacterium linked to both periodontitis and colorectal inflammation [152]. By precisely modulating dysbiotic microbial communities, phage-based interventions represent a novel preclinical approach toward restoring gut homoeostasis in IBD. Although not yet advanced to clinical trials, this precision strategy offers a compelling rationale for future interdisciplinary collaboration between dentistry and gastroenterology in targeting shared microbial drivers of mucosal inflammation.

### Clinical translation of collaborative management between dentistry and gastroenterology

Based on the oral-gut axis mechanism underlying periodontitis and IBD, the clinical translation of interdisciplinary collaboration between dentistry and gastroenterology relies on practical strategies that balance applicability and feasibility. First, a bidirectional referral system is essential: dentists should refer patients with moderate-to-severe periodontitis (especially those with elevated systemic inflammation) to gastroenterologists for intestinal mucosal screening and microbiota testing, while gastroenterologists should routinely arrange oral examinations for IBD patients to assess periodontal status. Second, an interdisciplinary combined monitoring system for inflammatory and immune biomarkers is required to evaluate the efficacy of co-therapy. For non-responsive patients, joint analysis by dentists and gastroenterologists—such as identifying incomplete eradication of oral pathogens or poor intestinal barrier repair—is critical for optimising interventions. Third, synergistic elimination of oral-derived pathogens is pivotal: basic periodontal therapy reduces oral pathogenic reservoirs, and targeted probiotics (guided by gut microbiota profiles) regulate intestinal microecology, preventing cross-site pathogen translocation that may occur with single-site treatment. Fourth, precision interventions targeting core pathobionts (e.g. *P. gingivalis, F. nucleatum*)—such as specific bacteriophages or vaccines—warrant animal and early-phase clinical studies. These studies should verify dual benefits (reducing oral pathogen load and ameliorating intestinal inflammation) to develop combined regimens of local periodontal targeting and intestinal microecological regulation. Finally, interdisciplinary patient education is indispensable: dentists should teach standardised oral hygiene practices (e.g. Bass technique, flossing) and highlight the impact of periodontal health on intestinal diseases, while gastroenterologists should guide diet and medication management and inform patients of the risk of oral infections exacerbating intestinal inflammation. Collectively, these strategies bridge basic research and clinical practice, providing a framework for the integrated management of periodontitis and IBD.

## Conclusion and perspectives

This review establishes a systematic theoretical framework for the periodontitis-IBD regulatory axis by integrating perspectives from microbial ecology, host immunology, and innovative therapeutics. Core mechanisms driving this crosstalk are elucidated, including pathogen translocation, the propagation of dysbiosis, and the pivotal roles of microbial metabolites and immune mediators. Beyond mechanistic insights, current evidence on bidirectional interventions targeting the oral-gut-bone axis is summarised. These interventions range from reciprocal therapeutic regimens and natural anti-inflammatory compounds to advanced strategies such as microbiota transplantation (both oral and faecal) and phage therapy. Collectively, these findings advocate for a paradigm shift toward an integrated dental-gastroenterological model for diagnosis and treatment.

Despite these advances, critical knowledge gaps persist that must be addressed to translate this framework into clinical practice. First, epidemiological data on the baseline prevalence of periodontitis-IBD comorbidity across diverse populations remain scarce; future studies are essential to define risk modifiers related to disease subtype, demographics, and lifestyle factors to quantify the true burden of this comorbidity. Second, mechanistic understanding remains limited regarding pathway specificity and inter-individual heterogeneity. Integrating multi-omics approaches will be crucial to map the ‘oral-gut axis’ and pinpoint key molecular nodes. Crucially, the dynamic evolution of periodontitis-related biomarkers must be leveraged as a predictive tool. Future research should focus on correlating stage-specific shifts in inflammatory mediators (e.g. TNF-*α*, IL-6, MMPs) and repair factors (e.g. IL-10, Tregs) with intestinal outcomes, thereby establishing a longitudinal monitoring system to evaluate the efficacy of oral-gut targeted therapies. Of particular urgency is deciphering how immune tolerance imbalance drives cross-organ inflammation and how circulating oral-derived cytokines reshape the intestinal immune microenvironment. Furthermore, high-resolution characterisation of core pathobionts (e.g. *P. gingivalis*, *F. nucleatum*) at the strain level is needed to clarify their colonisation patterns. Finally, deeper investigation into microbial metabolites and their impact on intestinal barrier integrity will refine the ‘microbiota-metabolite-host’ paradigm, ultimately informing targeted interventional trials where biomarker trajectories serve as key endpoints for ameliorating IBD outcomes through periodontal therapy.

## Data Availability

No datasets were generated or analysed during the current study.

## References

[1] GBD 2021 Oral Disorders Collaborators. Trends in the global, regional, and national burden of oral conditions from 1990 to 2021: a systematic analysis for the global burden of disease study 2021. Lancet. 2025;405(10482):897–910. doi: 10.1016/s0140-6736(24)02811-3 40024264

[2] Zilberstein NF, Engen PA, Swanson GR, et al. The bidirectional effects of periodontal disease and oral dysbiosis on gut inflammation in inflammatory bowel disease. J Crohns Colitis. 2025;19(4). doi: 10.1093/ecco-jcc/jjae162 PMC12041420 39447062

[3] Hajishengallis G, Chavakis T. Local and systemic mechanisms linking periodontal disease and inflammatory comorbidities. Nat Rev Immunol. 2021;21(7):426–440. doi: 10.1038/s41577-020-00488-6 33510490 PMC7841384

[4] Polizzi A, Nibali L, Tartaglia GM, et al. Impact of nonsurgical periodontal treatment on arterial stiffness outcomes related to endothelial dysfunction: a systematic review and meta-analysis. J Periodontol. 2025;96(4):330–345. doi: 10.1002/jper.24-0422 39549247 PMC12062727

[5] Polizzi A, Alibrandi A, Lo Giudice A, et al. Impact of periodontal microRNAs associated with alveolar bone remodeling during orthodontic tooth movement: a randomized clinical trial. J Transl Med. 2024;22(1):1155. doi: 10.1186/s12967-024-05933-x 39736760 PMC11684316

[6] Zhang Y, Bian C, Yu C, et al. Bidirectional association between oral diseases caused by plaque and the inflammatory bowel disease: a systematic review and meta-analysis. Jpn Dent Sci Rev. 2025;61:7–21. doi: 10.1016/j.jdsr.2025.02.001 40041882 PMC11876832

[7] Kunath BJ, De Rudder C, Laczny CC, et al. The oral-gut microbiome axis in health and disease. Nat Rev Microbiol. 2024;22(12):791–805. doi: 10.1038/s41579-024-01075-5 39039286

[8] Kaplan GG, Windsor JW. The four epidemiological stages in the global evolution of inflammatory bowel disease. Nat Rev Gastroenterol Hepatol. 2021;18(1):56–66. doi: 10.1038/s41575-020-00360-x 33033392 PMC7542092

[9] Tanwar H, Gnanasekaran JM, Allison D, et al. Unravelling the oral-gut axis: interconnection between periodontitis and inflammatory bowel disease, current challenges, and future perspective. J Crohns Colitis. 2024;18(8):1319–1341. doi: 10.1093/ecco-jcc/jjae028 38417137 PMC11324343

[10] Baima G, Muwalla M, Testa G, et al. Periodontitis prevalence and severity in inflammatory bowel disease: a case-control study. J Periodontol. 2023;94(3):313–322. doi: 10.1002/jper.22-0322 36111636

[11] Hou J, Yi Liu JX, Zhang H, et al. Sodium butyrate inhibits osteogenesis in human periodontal ligament stem cells by suppressing smad1 expression. BMC Oral Health. 2022 Jul 19;22(1):301. doi: 10.1186/s12903-022-02255-6 35854293 PMC9297574

[12] Zhao X, Liu J, Zhang C, et al. Porphyromonas gingivalis exacerbates ulcerative colitis via porphyromonas gingivalis peptidylarginine deiminase. Int J Oral Sci. 2021;13(1):31. doi: 10.1038/s41368-021-00136-2 34593756 PMC8484350

[13] Liu L, Liang L, Yang C, et al. Extracellular vesicles of fusobacterium nucleatum compromise intestinal barrier through targeting RIPK1-mediated cell death pathway. Gut Microbes. 2021;13(1):1–20. doi: 10.1080/19490976.2021.1902718 PMC8007154 33769187

[14] Chen Y, Chen Y, Cao P, et al. Fusobacterium nucleatum facilitates ulcerative colitis through activating IL-17F signaling to NF-κB via the upregulation of CARD3 expression. J Pathol. 2020;250(2):170–182. doi: 10.1002/path.5358 31610014

[15] Zhao YWR, Duan Y, Kong X, et al. Fusobacterium nucleatum-derived extracellular vesicles carrying virulence factor DNA trigger AIM2 inflammasome activation to facilitate UC progression. Cell Mol Biol Lett. Cell Mol Biol Lett.. 2025 Nov 17;30(1):138. doi: 10.1186/s11658-025-00817-4 41249954 PMC12625466

[16] Zhang Y, Sun Y, Hu Y, et al. Porphyromonas gingivalis msRNA P.G_45033 induces amyloid-β production by enhancing glycolysis and histone lactylation in macrophages. Int Immunopharmacol. 2023;121:110468. doi: 10.1016/j.intimp.2023.110468 37320870

[17] Zhao X, Zheng X, Wang Y, et al. Administration of porphyromonas gingivalis in pregnant mice enhances glycolysis and histone lactylation/ADAM17 leading to cleft palate in offspring. Int J Oral Sci. 2025;17(1):18. doi: 10.1038/s41368-025-00347-x 40075093 PMC11903673

[18] Shi X, Liu J, Lu Z, et al. Role of ferroptosis in porphyromonas gingivalis-induced impairment of epithelial junction. J Oral Microbiol. 2024;16(1):2334578. doi: 10.1080/20002297.2024.2334578 38562512 PMC10984227

[19] Wang Y, Wang L, Sun T, et al. Study of the inflammatory activating process in the early stage of fusobacterium nucleatum infected PDLSCs. Int J Oral Sci. 2023;15(1):8. doi: 10.1038/s41368-022-00213-0 36754953 PMC9908923

[20] Zhang X, Cheng S, Chen S, et al. Periodontitis-associated fusobacterium nucleatum promotes ulcerative colitis by ferroptosis-mediated gut barrier disruption. NPJ Biofilms Microbiomes. 2025;11(1):155. doi: 10.1038/s41522-025-00763-1 40783488 PMC12335446

[21] Zhou J, Yuan Z, Yang R, et al. Coaggregated E. Faecalis with F. Nucleatum regulated environmental stress responses and inflammatory effects. Appl Microbiol Biotechnol. 2024;108(1):336. doi: 10.1007/s00253-024-13172-9 38761182 PMC11102388

[22] Zhu J, Jiang Z, Yu F, et al. Integrated oral-gut microbiota therapy: a novel perspective on preventing bacterial translocation for systemic disease management. Front Cell Infect Microbiol. 2025;15:1641816. doi: 10.3389/fcimb.2025.1641816 40792109 PMC12336206

[23] Baima G, Ferrocino I, Del Lupo V, et al. Effect of periodontitis and periodontal therapy on oral and gut microbiota. J Dent Res. 2024;103(4):359–368. doi: 10.1177/00220345231222800 38362600

[24] Bao J, Li L, Zhang Y, et al. Periodontitis May induce gut microbiota dysbiosis via salivary microbiota. Int J Oral Sci. 2022;14(1):32. doi: 10.1038/s41368-022-00183-3 35732628 PMC9217941

[25] Qian J, Lu J, Huang Y, et al. Periodontitis salivary microbiota worsens colitis. J Dent Res. 2022;101(5):559–568. doi: 10.1177/00220345211049781 34796773

[26] Zhang YW, Wu Y, Liu XF, et al. Targeting the gut microbiota-related metabolites for osteoporosis: the inextricable connection of gut-bone axis. Ageing Res Rev. 2024;94:102196. doi: 10.1016/j.arr.2024.102196 38218463

[27] Li W, Liang H, Lin X, et al. A catalog of bacterial reference genomes from cultivated human oral bacteria. NPJ Biofilms Microbiomes. 2023;9(1):45. doi: 10.1038/s41522-023-00414-3 37400465 PMC10318035

[28] Curtis MA, Diaz PI, Van Dyke TE. The role of the microbiota in periodontal disease. Periodontol 2000. 2020;83(1):14–25. doi: 10.1111/prd.12296 32385883

[29] Procházková N, Falony G, Dragsted LO, et al. Advancing human gut microbiota research by considering gut transit time. Gut. 2023;72(1):180–191. doi: 10.1136/gutjnl-2022-328166 36171079 PMC9763197

[30] Zhu S, Han M, Liu S, et al. Composition and diverse differences of intestinal microbiota in ulcerative colitis patients. Front Cell Infect Microbiol. 2022;12:953962. doi: 10.3389/fcimb.2022.953962 36111238 PMC9468541

[31] Dinakaran V, Mandape SN, Shuba K, et al. Identification of specific oral and gut pathogens in full thickness colon of colitis patients: implications for colon motility. Front Microbiol. 2018;9:3220. doi: 10.3389/fmicb.2018.03220 30666239 PMC6330997

[32] Yamazaki K, Kamada N. Exploring the oral-gut linkage: interrelationship between oral and systemic diseases. Mucosal Immunol. 2024;17(1):147–153. doi: 10.1016/j.mucimm.2023.11.006 38007003 PMC11222583

[33] Kitamoto S, Nagao-Kitamoto H, Jiao Y, et al. The intermucosal connection between the mouth and gut in commensal pathobiont-driven colitis. Cell. 2020;182(2):447–462 e414. doi: 10.1016/j.cell.2020.05.048 32758418 PMC7414097

[34] Huang Y, Liao Y, Luo B, et al. Non-surgical periodontal treatment restored the gut microbiota and intestinal barrier in apolipoprotein E(-/-) mice with periodontitis. Front Cell Infect Microbiol. 2020;10:498. doi: 10.3389/fcimb.2020.00498 33072621 PMC7536370

[35] Elzayat H, Mesto G, Al-Marzooq F. Unraveling the impact of gut and oral microbiome on gut health in inflammatory bowel diseases. Nutrients. 2023;15(15):3377. doi: 10.3390/nu15153377 37571313 PMC10421146

[36] Imai J, Ichikawa H, Kitamoto S, et al. A potential pathogenic association between periodontal disease and Crohn's disease. JCI Insight. 2021;6(23). doi: 10.1172/jci.insight.148543 PMC8675195 34710061

[37] Zhou T, Xu W, Wang Q, et al. The effect of the “Oral-Gut” axis on periodontitis in inflammatory bowel disease: a review of microbe and immune mechanism associations. Front Cell Infect Microbiol. 2023;13:1132420. doi: 10.3389/fcimb.2023.1132420 36923589 PMC10008960

[38] Xiao X, Zhang X, Wang J, et al. Proton pump inhibitors alter gut microbiota by promoting oral microbiota translocation: a prospective interventional study. Gut. 2024;73(7):1098–1109. doi: 10.1136/gutjnl-2023-330883 38267200

[39] Shan Y, Lee M, Chang EB. The gut microbiome and inflammatory bowel diseases. Annu Rev Med. 2022;73:455–468. doi: 10.1146/annurev-med-042320-021020 34555295 PMC10012812

[40] Zheng Z, Jin W, Guo W, et al. Oral fusobacterium nucleatum exacerbates ulcerative colitis via the oral-gut axis: mechanisms and therapeutic implications. Front Cell Infect Microbiol. 2025;15:1564169. doi: 10.3389/fcimb.2025.1564169 40260115 PMC12009839

[41] Jiang SS, Chen YX, Fang JY. Fusobacterium nucleatum: ecology, pathogenesis and clinical implications. Nat Rev Microbiol. 2025;24:197–214. doi: 10.1038/s41579-025-01237-z 40983729

[42] Lu Y, Li Z, Peng X. Regulatory effects of oral microbe on intestinal microbiota and the illness. Front Cell Infect Microbiol. 2023;13:1093967. doi: 10.3389/fcimb.2023.1093967 36816583 PMC9928999

[43] Abdelbary MMH, Hatting M, Bott A, et al. The oral-gut axis: salivary and fecal microbiome dysbiosis in patients with inflammatory bowel disease. Front Cell Infect Microbiol. 2022;12:1010853. doi: 10.3389/fcimb.2022.1010853 36275026 PMC9585322

[44] Qi Y, Zang SQ, Wei J, et al. High-throughput sequencing provides insights into oral microbiota dysbiosis in association with inflammatory bowel disease. Genomics. 2021;113(1 Pt 2):664–676. doi: 10.1016/j.ygeno.2020.09.063 33010388

[45] Said HS, Suda W, Nakagome S, et al. Dysbiosis of salivary microbiota in inflammatory bowel disease and its association with oral immunological biomarkers. DNA Res. 2014;21(1):15–25. doi: 10.1093/dnares/dst037 24013298 PMC3925391

[46] Byrd KM, Gulati AS. The “Gum-Gut” axis in inflammatory bowel diseases: a hypothesis-driven review of associations and advances. Front Immunol. 2021;12:620124. doi: 10.3389/fimmu.2021.620124 33679761 PMC7933581

[47] Rautava J, Pinnell LJ, Vong L, et al. Oral microbiome composition changes in mouse models of colitis. J Gastroenterol Hepatol. 2015;30(3):521–527. doi: 10.1111/jgh.12713 25180790

[48] Santos AFP, Cervantes LCC, Panahipour L, et al. Proof-of-principle study suggesting potential anti-inflammatory activity of butyrate and propionate in periodontal cells. Int J Mol Sci. 2022;23(19):11006. doi: 10.3390/ijms231911006 36232340 PMC9570314

[49] Kimura I, Ichimura A, Ohue-Kitano R, et al. Free fatty acid receptors in health and disease. Physiol Rev. 2020;100(1):171–210. doi: 10.1152/physrev.00041.2018 31487233

[50] Hays KE, Pfaffinger JM, Ryznar R. The interplay between gut microbiota, short-chain fatty acids, and implications for host health and disease. Gut Microbes. 2024;16(1):2393270. doi: 10.1080/19490976.2024.2393270 39284033 PMC11407412

[51] Zeng X, Sun L, Xie H, et al. Lactobacillus johnsonii generates Cyclo(pro-trp) and promotes intestinal Ca(2+) absorption to alleviate CKD-SHPT. Adv Sci (Weinh). 2025;12(16):e2414678. doi: 10.1002/advs.202414678 39887665 PMC12021065

[52] Yang W, Yu T, Huang X, et al. Intestinal microbiota-derived short-chain fatty acids regulation of immune cell IL-22 production and gut immunity. Nat Commun. 2020;11(1):4457. doi: 10.1038/s41467-020-18262-6 32901017 PMC7478978

[53] Basic A, Dahlén G. Microbial metabolites in the pathogenesis of periodontal diseases: a narrative review. Front Oral Health. 2023;4:1210200. doi: 10.3389/froh.2023.1210200 37388417 PMC10300593

[54] Baima G, Dabdoub S, Thumbigere-Math V, et al. Multi-omics signatures of periodontitis and periodontal therapy on the oral and gut microbiome. J Periodontal Res. 2025;60(12):1237–1253. doi: 10.1111/jre.70055 41307322 PMC12881887

[55] Wang Q, Sun Y, Zhou T, et al. Gut microbiota-dependent trimethylamine n-oxide pathway contributes to the bidirectional relationship between intestinal inflammation and periodontitis. Front Cell Infect Microbiol. 2022;12:1125463. doi: 10.3389/fcimb.2022.1125463 36710972 PMC9880481

[56] Huang L, Huang L, Wang L, et al. Nonsurgical periodontal treatment improved the abnormal trimethylamine n-oxide metabolism in Apoe(-/-) mice with periodontitis. Biochim Biophys Acta Mol Basis Dis. 2025;1871(5):167752. doi: 10.1016/j.bbadis.2025.167752 40021025

[57] Wang N, Hao Y, Fu L. Trimethylamine-N-Oxide promotes osteoclast differentiation and bone loss via activating ROS-Dependent NF-κB signaling pathway. Nutrients. 2022;14(19):3955. doi: 10.3390/nu14193955 36235607 PMC9573743

[58] Chen Y, Yang C, Dai Q, et al. Gold-nanosphere mitigates osteoporosis through regulating TMAO metabolism in a gut microbiota-dependent manner. J Nanobiotechnology. 2023;21(1):125. doi: 10.1186/s12951-023-01872-9 37041523 PMC10088181

[59] Xiao HW, Cui M, Li Y, et al. Gut microbiota-derived indole 3-propionic acid protects against radiation toxicity via retaining acyl-CoA-binding protein. Microbiome. 2020;8(1):69. doi: 10.1186/s40168-020-00845-6 32434586 PMC7241002

[60] Zhao Y, Peng X, Wang Q, et al. Crosstalk between the neuroendocrine system and bone homeostasis. Endocr Rev. 2024;45(1):95–124. doi: 10.1210/endrev/bnad025 37459436

[61] Yang F, Yan Q, Wang Y, et al. AMP1-1 alleviates bone loss in weightless rats by reducing peripheral 5-HT content via the microbiota-gut-bone axis. Phytomedicine. 2025;139:156447. doi: 10.1016/j.phymed.2025.156447 39923429

[62] Agus A, Clément K, Sokol H. Gut microbiota-derived metabolites as central regulators in metabolic disorders. Gut. 2021;70(6):1174–1182. doi: 10.1136/gutjnl-2020-323071 33272977 PMC8108286

[63] Nakamura A, Kurihara S, Takahashi D, et al. Symbiotic polyamine metabolism regulates epithelial proliferation and macrophage differentiation in the colon. Nat Commun. 2021;12(1):2105. doi: 10.1038/s41467-021-22212-1 33833232 PMC8032791

[64] Bui TI, Gill AL, Mooney RA, et al. Modulation of gut microbiota metabolism in obesity-related type 2 diabetes reduces osteomyelitis severity. Microbiol Spectr. 2022;10(2):e0017022. doi: 10.1128/spectrum.00170-22 35315698 PMC9045376

[65] Yang Z, Zhang XW, Zhuo FF, et al. Allosteric activation of transglutaminase 2 via inducing an “Open” conformation for osteoblast differentiation. Adv Sci (Weinh). 2023;10(18):e2206533. doi: 10.1002/advs.202206533 37088726 PMC10288273

[66] Zhang J, Li X, Zhao K, et al. In vitro digestion and fermentation combined with microbiomics and metabolomics reveal the mechanism of superfine yak bone powder regulating lipid metabolism by altering human gut microbiota. Food Chem. 2023;410:135441. doi: 10.1016/j.foodchem.2023.135441 36652799

[67] Shim JA, Ryu JH, Jo Y, et al. The role of gut microbiota in T cell immunity and immune mediated disorders. Int J Biol Sci. 2023;19(4):1178–1191. doi: 10.7150/ijbs.79430 36923929 PMC10008692

[68] Engevik MA, Danhof HA, Auchtung J, et al. Fusobacteriumnucleatum adheres to clostridioides difficile via the RadD adhesin to enhance biofilm formation in intestinal mucus. Gastroenterology. 2021;160(4):1301–1314 e1308. doi: 10.1053/j.gastro.2020.11.034 33227279 PMC7956072

[69] Atarashi K, Suda W, Luo C, et al. Ectopic colonization of oral bacteria in the intestine drives T(H)1 cell induction and inflammation. Science. 2017;358(6361):359–365. doi: 10.1126/science.aan4526 29051379 PMC5682622

[70] Wang N, Fang JY. Fusobacterium nucleatum, a key pathogenic factor and microbial biomarker for colorectal cancer. Trends Microbiol. 2023;31(2):159–172. doi: 10.1016/j.tim.2022.08.010 36058786

[71] Brennan CA, Clay SL, Lavoie SL, et al. Fusobacterium nucleatum drives a pro-inflammatory intestinal microenvironment through metabolite receptor-dependent modulation of IL-17 expression. Gut Microbes. 2021;13(1):1987780. doi: 10.1080/19490976.2021.1987780 34781821 PMC8604392

[72] Lorenzo J. From the gut to bone: connecting the gut microbiota with Th17 T lymphocytes and postmenopausal osteoporosis. J Clin Invest. 2021;131(5). doi: 10.1172/jci146619 PMC7919711 33645543

[73] Yuan X, Zhou F, Wang H, et al. Systemic antibiotics increase microbiota pathogenicity and oral bone loss. Int J Oral Sci. 2023;15(1):4. doi: 10.1038/s41368-022-00212-1 36631439 PMC9834248

[74] Danne C, Skerniskyte J, Marteyn B, et al. Neutrophils: from IBD to the gut microbiota. Nat Rev Gastroenterol Hepatol. 2024;21(3):184–197. doi: 10.1038/s41575-023-00871-3 38110547

[75] Wang A, Zhai Z, Ding Y, et al. The oral-gut microbiome axis in inflammatory bowel disease: from inside to insight. Front Immunol. 2024;15:1430001. doi: 10.3389/fimmu.2024.1430001 39131163 PMC11310172

[76] Xiang B, Hu J, Zhang M, et al. The involvement of oral bacteria in inflammatory bowel disease. Gastroenterol Rep (Oxf). 2024;12:goae076. doi: 10.1093/gastro/goae076 39188957 PMC11346772

[77] Wang F, Zhou F, Peng J, et al. Macrophage Tim-3 maintains intestinal homeostasis in DSS-induced colitis by suppressing neutrophil necroptosis. Redox Biol. 2024;70:103072. doi: 10.1016/j.redox.2024.103072 38330550 PMC10865407

[78] Cui G, Fan Q, Li Z, et al. Evaluation of anti-TNF therapeutic response in patients with inflammatory bowel disease: current and novel biomarkers. EBioMedicine. 2021;66:103329. doi: 10.1016/j.ebiom.2021.103329 33862588 PMC8054158

[79] Elhag DA, Kumar M, Saadaoui M, et al. Inflammatory bowel disease treatments and predictive biomarkers of therapeutic response. Int J Mol Sci. 2022;23(13):6966. doi: 10.3390/ijms23136966 35805965 PMC9266456

[80] Xie J, Li Q, Haesebrouck F, et al. The tremendous biomedical potential of bacterial extracellular vesicles. Trends Biotechnol. 2022;40(10):1173–1194. doi: 10.1016/j.tibtech.2022.03.005 35581020

[81] Xie J, Haesebrouck F, Van Hoecke L, et al. Bacterial extracellular vesicles: an emerging avenue to tackle diseases. Trends Microbiol. 2023;31(12):1206–1224. doi: 10.1016/j.tim.2023.05.010 37330381

[82] Kim HY, Song MK, Gho YS, et al. Extracellular vesicles derived from the periodontal pathogen filifactor alocis induce systemic bone loss through toll-like receptor 2. J Extracell Vesicles. 2021;10(12):e12157. doi: 10.1002/jev2.12157 34648247 PMC8516034

[83] Catalan EA, Seguel-Fuentes E, Fuentes B, et al. Oral pathobiont-derived outer membrane vesicles in the oral-gut axis. Int J Mol Sci. 2024;25(20):11141. doi: 10.3390/ijms252011141 39456922 PMC11508520

[84] Jones E, Stentz R, Telatin A, et al. The origin of plasma-derived bacterial extracellular vesicles in healthy individuals and patients with inflammatory bowel disease: a pilot study. Genes (Basel). 2021;12(10):1636. doi: 10.3390/genes12101636 34681030 PMC8535827

[85] Deo P, Chow SH, Han ML, et al. Mitochondrial dysfunction caused by outer membrane vesicles from gram-negative bacteria activates intrinsic apoptosis and inflammation. Nat Microbiol. 2020;5(11):1418–1427. doi: 10.1038/s41564-020-0773-2 32807891

[86] Ahmadi Badi S, Moshiri A, Ettehad Marvasti F, et al. Extraction and evaluation of outer membrane vesicles from two important gut microbiota members, bacteroides fragilis and bacteroides thetaiotaomicron. Cell J. 2020;22(3):344–349. doi: 10.22074/cellj.2020.6499 31863660 PMC6947009

[87] Chen CY, Rao SS, Yue T, et al. Glucocorticoid-induced loss of beneficial gut bacterial extracellular vesicles is associated with the pathogenesis of osteonecrosis. Sci Adv. 2022;8(15):eabg8335. doi: 10.1126/sciadv.abg8335 35417243 PMC9007505

[88] Liu JH, Chen CY, Liu ZZ, et al. Extracellular vesicles from child gut microbiota enter into bone to preserve bone mass and strength. Adv Sci (Weinh). 2021;8(9):2004831. doi: 10.1002/advs.202004831 33977075 PMC8097336

[89] Jing F, Zhang J, Zhang H, et al. Unlocking the multifaceted molecular functions and diverse disease implications of lactylation. Biol Rev Camb Philos Soc. 2025;100(1):172–189. doi: 10.1111/brv.13135 39279350

[90] Zhang D, Tang Z, Huang H, et al. Metabolic regulation of gene expression by histone lactylation. Nature. 2019;574(7779):575–580. doi: 10.1038/s41586-019-1678-1 31645732 PMC6818755

[91] Qiqiang L, Huanxin M, Xuejun G. Longitudinal study of volatile fatty acids in the gingival crevicular fluid of patients with periodontitis before and after nonsurgical therapy. J Periodontal Res. 2012;47(6):740–749. doi: 10.1111/j.1600-0765.2012.01489.x 22594616

[92] Ishikawa T, Sasaki D, Aizawa R, et al. The role of lactic acid on wound healing, cell growth, cell cycle kinetics, and gene expression of cultured junctional epithelium cells in the pathophysiology of periodontal disease. Pathogens. 2021;10(11):1507. doi: 10.3390/pathogens10111507 34832662 PMC8620665

[93] Weng Y, Wang H, Li L, et al. Trem2 mediated syk-dependent ROS amplification is essential for osteoclastogenesis in periodontitis microenvironment. Redox Biol. 2021;40:101849. doi: 10.1016/j.redox.2020.101849 33486152 PMC7823053

[94] Irizarry-Caro RA, McDaniel MM, Overcast GR, et al. TLR signaling adapter BCAP regulates inflammatory to reparatory macrophage transition by promoting histone lactylation. Proc Natl Acad Sci U S A. 2020;117(48):30628–30638. doi: 10.1073/pnas.2009778117 33199625 PMC7720107

[95] Lopez Krol A, Nehring HP, Krause FF, et al. Lactate induces metabolic and epigenetic reprogramming of pro-inflammatory Th17 cells. EMBO Rep. 2022;23(12):e54685. doi: 10.15252/embr.202254685 36215678 PMC9724659

[96] Sun S, Xu X, Liang L, et al. Lactic acid-producing probiotic saccharomyces cerevisiae attenuates ulcerative colitis via suppressing macrophage pyroptosis and modulating gut microbiota. Front Immunol. 2021;12:777665. doi: 10.3389/fimmu.2021.777665 34899735 PMC8652295

[97] Sczepanik FSC, Grossi ML, Casati M, et al. Periodontitis is an inflammatory disease of oxidative stress: we should treat it that way. Periodontol 2000. 2020;84(1):45–68. doi: 10.1111/prd.12342 32844417

[98] Buduneli N, Bıyıkoğlu B, Kinane DF. Utility of gingival crevicular fluid components for periodontal diagnosis. Periodontol 2000. 2024;95(1):156–175. doi: 10.1111/prd.12595 39004819

[99] Aytekin Z, Arabacı T, Toraman A, et al. Immune modulatory and antioxidant effects of locally administrated vitamin C in experimental periodontitis in rats. Acta Odontol Scand. 2020;78(6):425–432. doi: 10.1080/00016357.2020.1734656 32157939

[100] Lee M, Chang EB. Inflammatory bowel diseases (IBD) and the microbiome-searching the crime scene for clues. Gastroenterology. 2021;160(2):524–537. doi: 10.1053/j.gastro.2020.09.056 33253681 PMC8098834

[101] Ye R, Guo Q, Huang J, et al. Eucommia ulmoides polysaccharide modified nano-selenium effectively alleviated DSS-induced colitis through enhancing intestinal mucosal barrier function and antioxidant capacity. J Nanobiotechnology. 2023;21(1):222. doi: 10.1186/s12951-023-01965-5 37438752 PMC10337189

[102] Shandilya S, Kumar S, Kumar Jha N, et al. Interplay of gut microbiota and oxidative stress: perspective on neurodegeneration and neuroprotection. J Adv Res. 2022;38:223–244. doi: 10.1016/j.jare.2021.09.005 35572407 PMC9091761

[103] Dixon SJ, Pratt DA. Ferroptosis: a flexible constellation of related biochemical mechanisms. Mol Cell. 2023;83(7):1030–1042. doi: 10.1016/j.molcel.2023.03.005 36977413 PMC10081971

[104] Sui Y, Jiang R, Niimi M, et al. Gut bacteria exacerbates TNBS-induced colitis and kidney injury through oxidative stress. Redox Biol. 2024;72:103140. doi: 10.1016/j.redox.2024.103140 38593629 PMC11016804

[105] Han Y, Sun Y, Shu C, et al. Hepcidin-regulated iron metabolism disorders in patients with stage III/IV periodontitis. J Dent Sci. 2025;20(2):995–1001. doi: 10.1016/j.jds.2024.10.028 40224112 PMC11993015

[106] Dou J, Liu X, Yang L, et al. Ferroptosis interaction with inflammatory microenvironments: mechanism, biology, and treatment. Biomed Pharmacother. 2022;155:113711. doi: 10.1016/j.biopha.2022.113711 36126457

[107] Zhu J, Wang X, Su Y, et al. Multifunctional nanolocks with GSH as the key for synergistic ferroptosis and anti-chemotherapeutic resistance. Biomaterials. 2022;288:121704. doi: 10.1016/j.biomaterials.2022.121704 35948496

[108] Zhao X, Zhang J, Zhang W, et al. A chiral fluorescent Ir(iii) complex that targets the GPX4 and ErbB pathways to induce cellular ferroptosis. Chem Sci. 2023;14(5):1114–1122. doi: 10.1039/d2sc06171f 36756328 PMC9891362

[109] Xu M, Tao J, Yang Y, et al. Ferroptosis involves in intestinal epithelial cell death in ulcerative colitis. Cell Death Dis. 2020;11(2):86. doi: 10.1038/s41419-020-2299-1 32015337 PMC6997394

[110] Ji X, Ma S, Sun X, et al. Analysis of ferroptosis-associated genes in Crohn's disease based on bioinformatics. Front Med (Lausanne). 2022;9:1058076. doi: 10.3389/fmed.2022.1058076 36714107 PMC9881725

[111] Huang W, Aabed N, Shah YM. Reactive oxygen species and ferroptosis at the nexus of inflammation and colon cancer. Antioxid Redox Signal. 2023;39(7-9):551–568. doi: 10.1089/ars.2023.0246 36792928 PMC10517337

[112] Qi X, Zhang Y, Guo H, et al. Mechanism and intervention measures of iron side effects on the intestine. Crit Rev Food Sci Nutr. 2020;60(12):2113–2125. doi: 10.1080/10408398.2019.1630599 31232087

[113] Alemany-Cosme E, Sáez-González E, Moret I, et al. Oxidative stress in the pathogenesis of Crohn's disease and the interconnection with immunological response, microbiota, external environmental factors, and epigenetics. Antioxidants (Basel). 2021;10(1):64. doi: 10.3390/antiox10010064 33430227 PMC7825667

[114] Tao H, Li W, Zhang W, et al. Urolithin A suppresses RANKL-induced osteoclastogenesis and postmenopausal osteoporosis by, suppresses inflammation and downstream NF-κB activated pyroptosis pathways. Pharmacol Res. 2021;174:105967. doi: 10.1016/j.phrs.2021.105967 34740817

[115] Zhang YW, Cao MM, Li YJ, et al. Fecal microbiota transplantation ameliorates bone loss in mice with ovariectomy-induced osteoporosis via modulating gut microbiota and metabolic function. J Orthop Translat. 2022;37:46–60. doi: 10.1016/j.jot.2022.08.003 36196151 PMC9520092

[116] Yu M, Malik Tyagi A, Li JY, et al. PTH induces bone loss via microbial-dependent expansion of intestinal TNF(+) T cells and Th17 cells. Nat Commun. 2020;11(1):468. doi: 10.1038/s41467-019-14148-4 31980603 PMC6981196

[117] Li J, Zhang Z, Tang J, et al. Emerging roles of nerve-bone axis in modulating skeletal system. Med Res Rev. 2024;44(4):1867–1903. doi: 10.1002/med.22031 38421080

[118] Zhao Z, Ning J, Bao XQ, et al. Fecal microbiota transplantation protects rotenone-induced Parkinson's disease mice via suppressing inflammation mediated by the lipopolysaccharide-TLR4 signaling pathway through the microbiota-gut-brain axis. Microbiome. 2021;9(1):226. doi: 10.1186/s40168-021-01107-9 34784980 PMC8597301

[119] Ota M, Nagafuchi Y, Hatano H, et al. Dynamic landscape of immune cell-specific gene regulation in immune-mediated diseases. Cell. 2021;184(11):3006–3021.e3017. doi: 10.1016/j.cell.2021.03.056 33930287

[120] Estevinho MM, Yuan Y, Rodríguez-Lago I, et al. Efficacy and safety of probiotics in IBD: an overview of systematic reviews and updated meta-analysis of randomized controlled trials. United European Gastroenterol J. 2024;12(7):960–981. doi: 10.1002/ueg2.12636 PMC11497663 39106167

[121] Wei Y, Fu J, Wu W, et al. Quercetin prevents oxidative stress-induced injury of periodontal ligament cells and alveolar bone loss in periodontitis. Drug Des Devel Ther. 2021;15:3509–3522. doi: 10.2147/dddt.S315249 PMC8366957 34408403

[122] He Z, Zhang X, Song Z, et al. Quercetin inhibits virulence properties of porphyromas gingivalis in periodontal disease. Sci Rep. 2020;10(1):18313. doi: 10.1038/s41598-020-74977-y 33110205 PMC7591570

[123] An Y, Zhao R, Liu W, et al. Quercetin through miR-147-5p/Clip3 axis reducing Th17 cell differentiation to alleviate periodontitis. Regen Ther. 2024;27:496–505. doi: 10.1016/j.reth.2024.04.016 38756701 PMC11096707

[124] Hu H, Yao Y, Liu F, et al. Integrated microbiome and metabolomics revealed the protective effect of baicalin on alveolar bone inflammatory resorption in aging. Phytomedicine. 2024;124:155233. doi: 10.1016/j.phymed.2023.155233 38181526

[125] Zhang L, Miao C, Wang Z, et al. Preparation and characterisation of baicalin magnesium and its protective effect in ulcerative colitis via gut microbiota-bile acid axis modulation. Phytomedicine. 2024;126:155416. doi: 10.1016/j.phymed.2024.155416 38394726

[126] Huang Y, Ge R, Qian J, et al. Lacticaseibacillus rhamnosus GG improves periodontal bone repair via gut-blood axis in hyperlipidemia. J Dent Res. 2024;103(3):253–262. doi: 10.1177/00220345231217402 38197171

[127] Wu J, Zhou B, Pang X, et al. Clostridium butyricum, a butyrate-producing potential probiotic, alleviates experimental colitis through epidermal growth factor receptor activation. Food Funct. 2022;13(13):7046–7061. doi: 10.1039/d2fo00478j 35678197

[128] Zhou J, Li M, Chen Q, et al. Programmable probiotics modulate inflammation and gut microbiota for inflammatory bowel disease treatment after effective oral delivery. Nat Commun. 2022;13(1):3432. doi: 10.1038/s41467-022-31171-0 35701435 PMC9198027

[129] Zhang Z, Pan Y, Guo Z, et al. An olsalazine nanoneedle-embedded inulin hydrogel reshapes intestinal homeostasis in inflammatory bowel disease. Bioact Mater. 2024;33:71–84. doi: 10.1016/j.bioactmat.2023.10.028 38024237 PMC10658185

[130] Paramsothy S, Nielsen S, Kamm MA, et al. Specific bacteria and metabolites associated with response to fecal microbiota transplantation in patients with ulcerative colitis. Gastroenterology. 2019;156(5):1440–1454 e1442. doi: 10.1053/j.gastro.2018.12.001 30529583

[131] Yang Y, Zheng X, Wang Y, et al. Human fecal microbiota transplantation reduces the susceptibility to dextran sulfate sodium-induced germ-free mouse colitis. Front Immunol. 2022;13:836542. doi: 10.3389/fimmu.2022.836542 35237276 PMC8882623

[132] Lima SF, Gogokhia L, Viladomiu M, et al. Transferable immunoglobulin A-Coated odoribacter splanchnicus in responders to fecal microbiota transplantation for ulcerative colitis limits colonic inflammation. Gastroenterology. 2022;162(1):166–178. doi: 10.1053/j.gastro.2021.09.061 34606847 PMC8678328

[133] Huang C, Mei Q, Lou L, et al. Ulcerative colitis in response to fecal microbiota transplantation via modulation of gut microbiota and Th17/Treg cell balance. Cells. 2022;11(11):1851. doi: 10.3390/cells11111851 35681546 PMC9180439

[134] Isola G, Annunziata M, Angjelova A, et al. Effectiveness of two supportive periodontal care protocols and outcome predictors during periodontitis: a randomized controlled trial. J Periodontol. 2025. doi: 10.1002/jper.70007 PMC13111776 41201881

[135] Rocco S, Tempesta AA, Aluisio GV, et al. Antibacterial and cytotoxic effects of chlorhexidine combined with sodium DNA on oral microorganisms: an in vitro study using dictyostelium discoideum. J Oral Microbiol. 2025;17(1):2595797. doi: 10.1080/20002297.2025.2595797 41376801 PMC12687888

[136] de Oliveira AM, Lourenço TGB, Colombo APV. Impact of systemic probiotics as adjuncts to subgingival instrumentation on the oral-gut microbiota associated with periodontitis: a randomized controlled clinical trial. J Periodontol. 2022;93(1):31–44. doi: 10.1002/jper.21-0078 34028826

[137] Bamias G, Menghini P, Pizarro TT, et al. Targeting TL1A and DR3: the new frontier of anti-cytokine therapy in IBD. Gut. 2025;74(4):652–668. doi: 10.1136/gutjnl-2024-332504 39266053 PMC11885054

[138] Juiz PJL, Ferreira LTB, Pires EA, et al. Patent mining on the use of antioxidant phytochemicals in the technological development for the prevention and treatment of periodontitis. Antioxidants (Basel). 2024;13(5):566. doi: 10.3390/antiox13050566 38790671 PMC11117607

[139] Li X, Yu M, Zhu Z, et al. Oral delivery of infliximab using nano-in-microparticles for the treatment of inflammatory bowel disease. Carbohydr Polym. 2021;273:118556. doi: 10.1016/j.carbpol.2021.118556 34560967

[140] Ausenda F, Barbera E, Cotti E, et al. Clinical, microbiological and immunological short, medium and long-term effects of different strains of probiotics as an adjunct to non-surgical periodontal therapy in patients with periodontitis. Systematic review with meta-analysis. Jpn Dent Sci Rev. 2023;59:62–103. doi: 10.1016/j.jdsr.2023.02.001 36915665 PMC10006838

[141] Luo X, Kong Q, Wang Y, et al. Colonization of clostridium butyricum in rats and its effect on intestinal microbial composition. Microorganisms. 2021;9(8):1573. doi: 10.3390/microorganisms9081573 34442652 PMC8401576

[142] Li P, Ji B, Luo H, et al. One-year supplementation with lactobacillus reuteri ATCC PTA 6475 counteracts a degradation of gut microbiota in older women with low bone mineral density. NPJ Biofilms Microbiomes. 2022;8(1):84. doi: 10.1038/s41522-022-00348-2 36261538 PMC9581899

[143] Yang Q, Zhu Y, Jian X, et al. Targeting enterobacter cloacae attenuates osteolysis by reducing ammonium in multiple myeloma. Blood. 2025;145(17):1876–1889. doi: 10.1182/blood.2024025694 39786379

[144] Wang Z, Chen K, Wu C, et al. An emerging role of prevotella histicola on estrogen deficiency-induced bone loss through the gut microbiota-bone axis in postmenopausal women and in ovariectomized mice. AJCN. 2021;114(4):1304–1313. doi: 10.1093/ajcn/nqab194 34113963

[145] Hughes RL, Alvarado DA, Swanson KS, et al. The prebiotic potential of inulin-type fructans: a systematic review. Adv Nutr. 2022;13(2):492–529. doi: 10.1093/advances/nmab119 34555168 PMC8970830

[146] Nath S, Zilm P, Jamieson L, et al. Development and characterization of an oral microbiome transplant among Australians for the treatment of dental caries and periodontal disease: a study protocol. PLoS One. 2021;16(11):e0260433. doi: 10.1371/journal.pone.0260433 34843568 PMC8629173

[147] Beikler T, Kaymaz K, Chan Y, et al. Oral microbiota transplant in dogs with naturally occurring periodontitis. J Dent Res. 2021;100(7):764–770. doi: 10.1177/0022034521995423 33733913 PMC8217902

[148] Lindo CE, Sebastian J, Kuntjoro KN, et al. Microbiota transplantation as an adjunct to standard periodontal treatment in periodontal disease: a systematic review. Medicina (Kaunas). 2024;60(4):672. doi: 10.3390/medicina60040672 38674317 PMC11051950

[149] Haifer C, Paramsothy S, Kaakoush NO, et al. Lyophilised oral faecal microbiota transplantation for ulcerative colitis (LOTUS): a randomised, double-blind, placebo-controlled trial. Lancet Gastroenterol Hepatol. 2022;7(2):141–151. doi: 10.1016/s2468-1253(21)00400-3 34863330

[150] Chu ND, Crothers JW, Nguyen LTT, et al. Dynamic colonization of microbes and their functions after fecal microbiota transplantation for inflammatory bowel disease. mBio. 2021;12(4):e0097521. doi: 10.1128/mBio.00975-21 34281401 PMC8406238

[151] Zhao Y, Zhu M, Ling Y, et al. A DNA nanopatch-bacteriophage system targeting streptococcus gallolyticus for inflammatory bowel disease treatment and colorectal cancer prevention. Adv Mater. 2025;37(11):e2417334. doi: 10.1002/adma.202417334 39924920

[152] Zheng DW, Dong X, Pan P, et al. Phage-guided modulation of the gut microbiota of mouse models of colorectal cancer augments their responses to chemotherapy. Nat Biomed Eng. 2019;3(9):717–728. doi: 10.1038/s41551-019-0423-2 31332342

