# Correction

**DOI:** 10.1080/20002297.2026.2659411

**Published:** 2026-04-14

**Article title**: Microbial mechanisms and therapeutic interventions in the periodontitis-inflammatory bowel disease axis: a comprehensive review

**Authors**: Lv, W., Hu, H., Huang, Y., Yang, J., Li, Y., He, J., Wang, K., Liu, Y., & Wang, Q.

**Journal**: *Journal of Oral Microbiology*

**Bibliometrics:** Volume 18, Number 1, pages 1—22

**DOI:** http://doi.org/10.1080/20002297.2026.2656084

This article was first published with errors in Table 1 and Table 2:

The Table 1 and Table 2 has been updated as per updated source.

The article has been republished with errors corrected.

